# Supplementary figures and images for: Glyphosate inhibits melanization and increases susceptibility to infection in insects
Source: PLoS Biol. 2021 May 12;19(5):e3001182. doi: 10.1371/journal.pbio.3001182 (PMC8115815; doi:10.1371/journal.pbio.3001182)

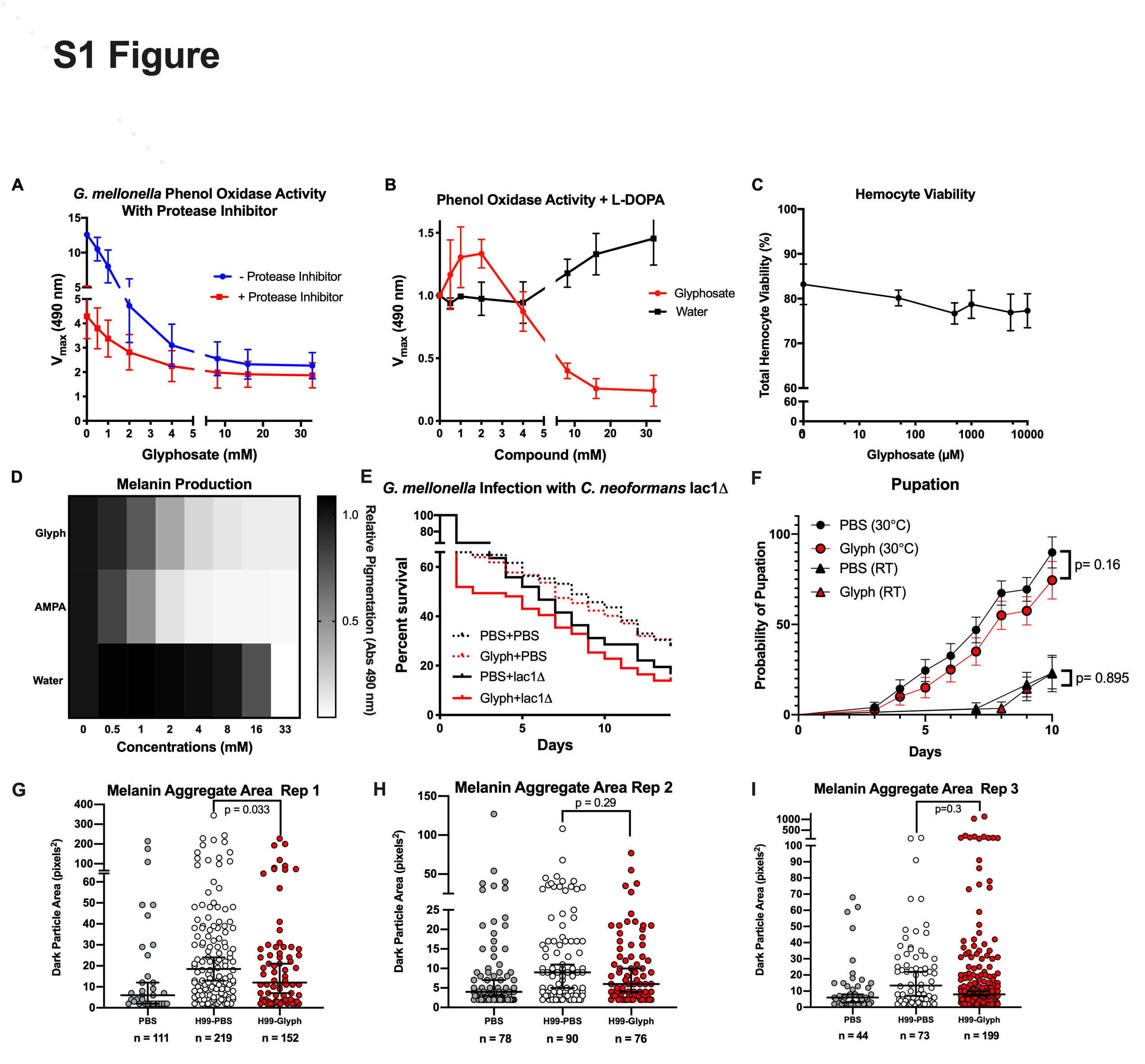

Supplement: S1 Fig — (A) Broad-spectrum protease inhibitor (cOmplete, Roche, Basel, Switzerland) was added to G. mellonella hemolymph to prevent the activation of new phenoloxidase and to control for any impact that glyphosate may have on phenoloxidase activation cascade, cell viability, and gene expression. The general trend remains the same that glyphosate inhibits phenoloxidase activity with and without protease inhibitor, albeit lower with protease inhibitor due to the lower concentration of activated enzyme. (B) Phenoloxidase activity was assessed using exogenous L-DOPA for 1 batch of G. mellonella, during these experiments, the lower concentration of glyphosate resulted in increased phenoloxidase activity as compared to the control. This suggests that there may be some cellular regulation of phenoloxidase induced by glyphosate. It is possible that the doses of glyphosate tested elicit some cellular response that increases phenoloxidase expression, secretion, and/or activation as a feedback/hormesis-like response to the reduced melanin production. These data represent 3 independent replicates, but this pattern of enzymatic activity as a function of glyphosate concentration was not seen in subsequent batches of larvae. (C) Hemocyte viability was not dramatically affected by concentrations of glyphosate ranging from 100 μM to 10 mM, indicating that our data are likely not artifacts of cytotoxic concentrations of glyphosate. Error bars in (A–C) represent ±SD. (D) AMPA, a major metabolite of glyphosate, inhibits tyrosinase-mediated melanization similar to glyphosate. Grayscale bars represent mean absorbance at 490 nm relative to no compound control. The darker colors correspond to increased pigment formation. (E) Larvae treated with glyphosate and subsequently infected with lac1Δ mutant C. neoformans strain showed a similar pattern of increased susceptibility as the wild-type H99, although the differences in susceptibility with the lac1Δ infected larvae are not statistically significan [file pbio.3001182.s001.tiff]

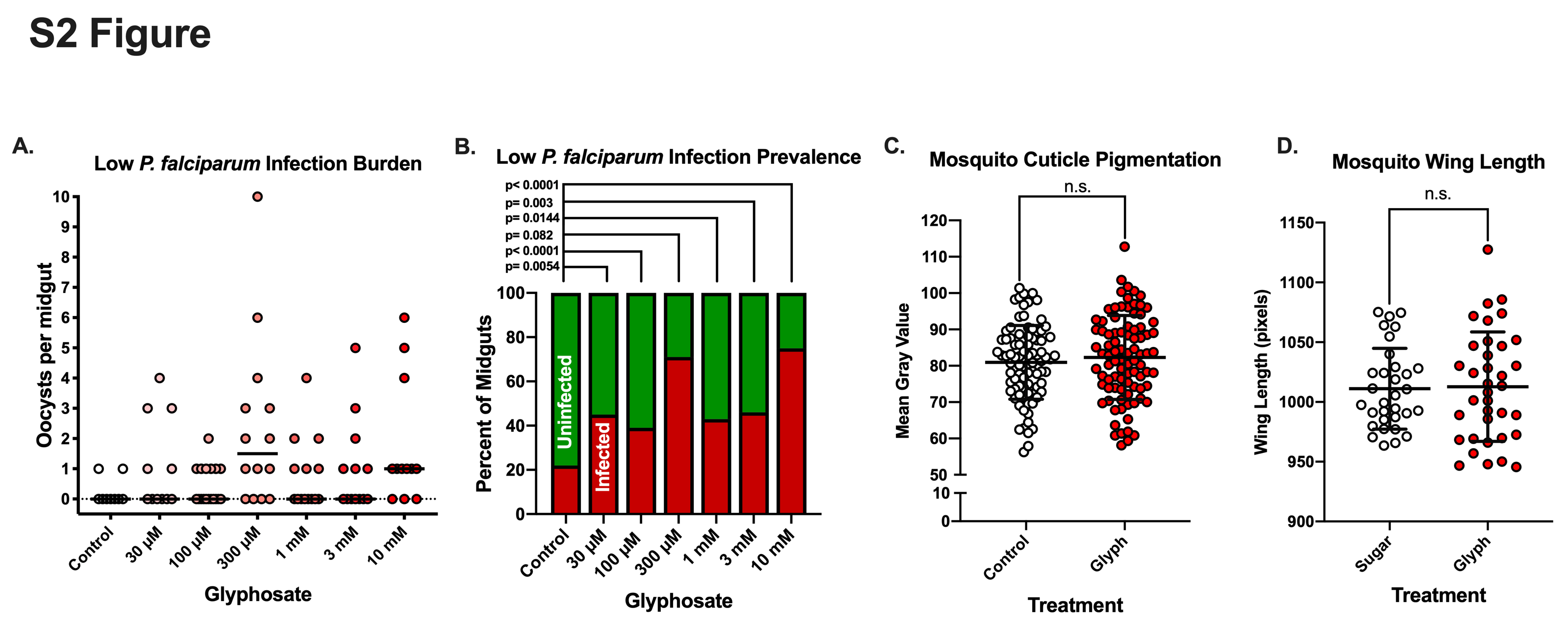

Supplement: S2 Fig — (A). Oocyst count per midgut for mosquitoes treated with or without glyphosate and infected with high-passage P. falciparum gametocyte culture, resulting in a low efficiency infection. Data represent 1 biological replicate. Dotted black line indicates y = 0. Black lines for each condition indicate median oocyst count per midgut. We have chosen not to include the data from this replicate in the data shown in Fig 6, because the results from this one-off replicate appear due to poorly infectious parasite culture. Additionally, it is difficult to make comparisons using the low infection burden of the control group with the treatment groups, as well other replicates with higher oocyst burdens. (B) Infection prevalence (percent midguts with at least 1 oocyst) from the experiment described in (A). Fisher exact test performed for each condition individually compared to control and corrected for multiple comparisons using the Bonferroni method. (C) A total of 5 days of 1 mM glyphosate treatment in adult female mosquitoes does not influence the abdomen’s cuticular darkness as measured by mean gray value with 0 being pure black and 255 being pure white. Data representative of 2 biological replicates with 88 mosquitoes measured per condition. (D) Wing length, as a proxy for body mass and size, is not affected by 5 days of glyphosate treatment. Data representative of a single biological replicates with 32–36 mosquitoes measured per condition. Line and error bar represent mean ± SD in (C, D). Unpaired t test performed to determine statistical significance in (C, D). All statistical analyses performed using GraphPad Prism version 8.4.3 for Mac OS, GraphPad Software, San Diego, California, USA, www.graphpad.com. For underlying data, please see Data Availability section and/or S1 Table. Glyph, glyphosate; n.s., not significant. (TIFF) [file pbio.3001182.s002.tiff]

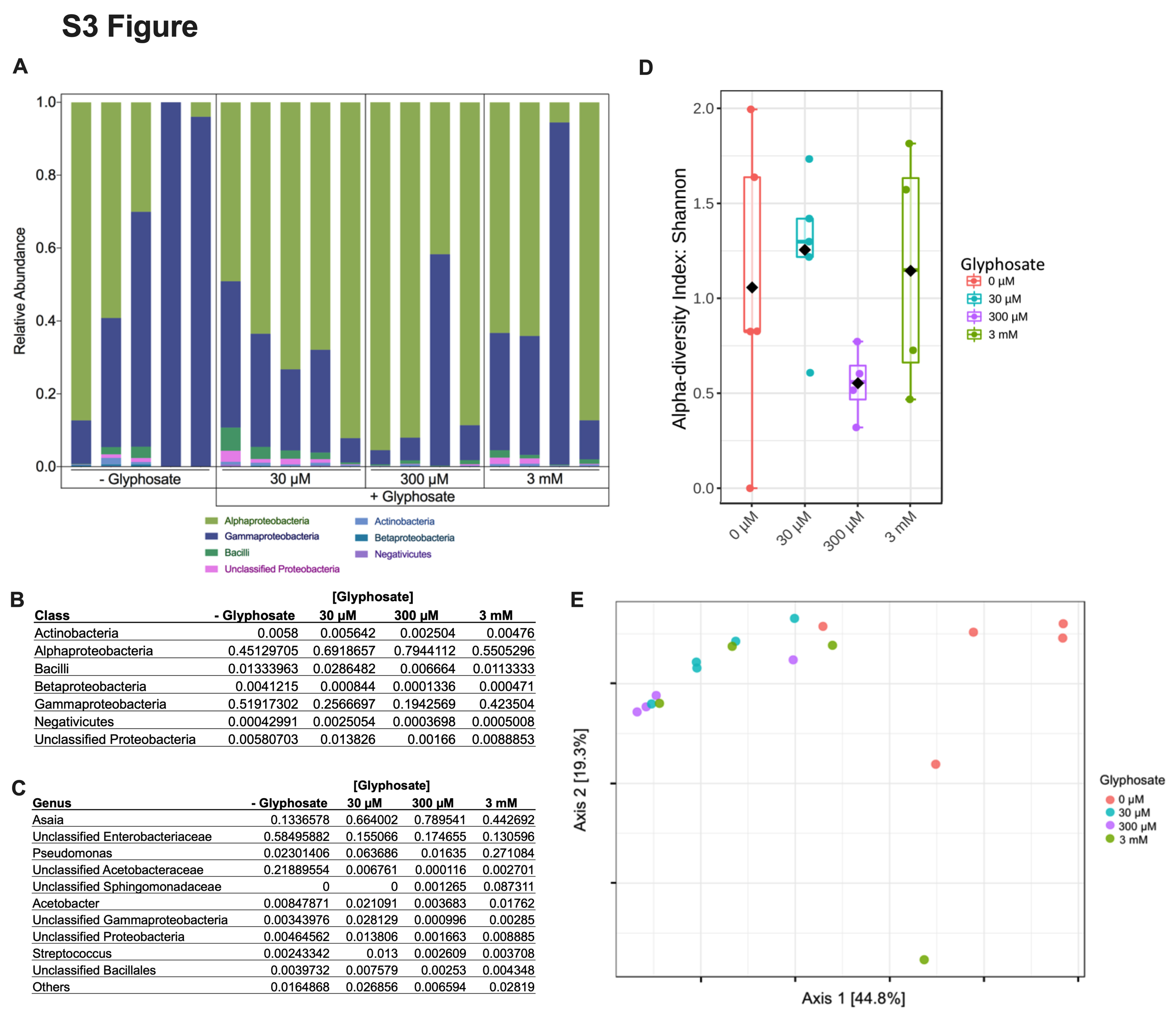

Supplement: S3 Fig — (A) At the class level, glyphosate leads to an enrichment of Alphaproteobacteria and a depletion in Gammaproteobacteria. Tables showing the relative abundance of bacterial classes (B) and individual bacterial genera (C) following glyphosate treatment. (D) Alpha diversity does not follow a distinctive pattern with increasing glyphosate dose. (E) Glyphosate-treated and control-treated microbiota cluster separately in ordination space, but the clusters are not dose dependent. For underlying data, please see Data Availability section and/or S1 Table. (TIFF) [file pbio.3001182.s003.tiff]

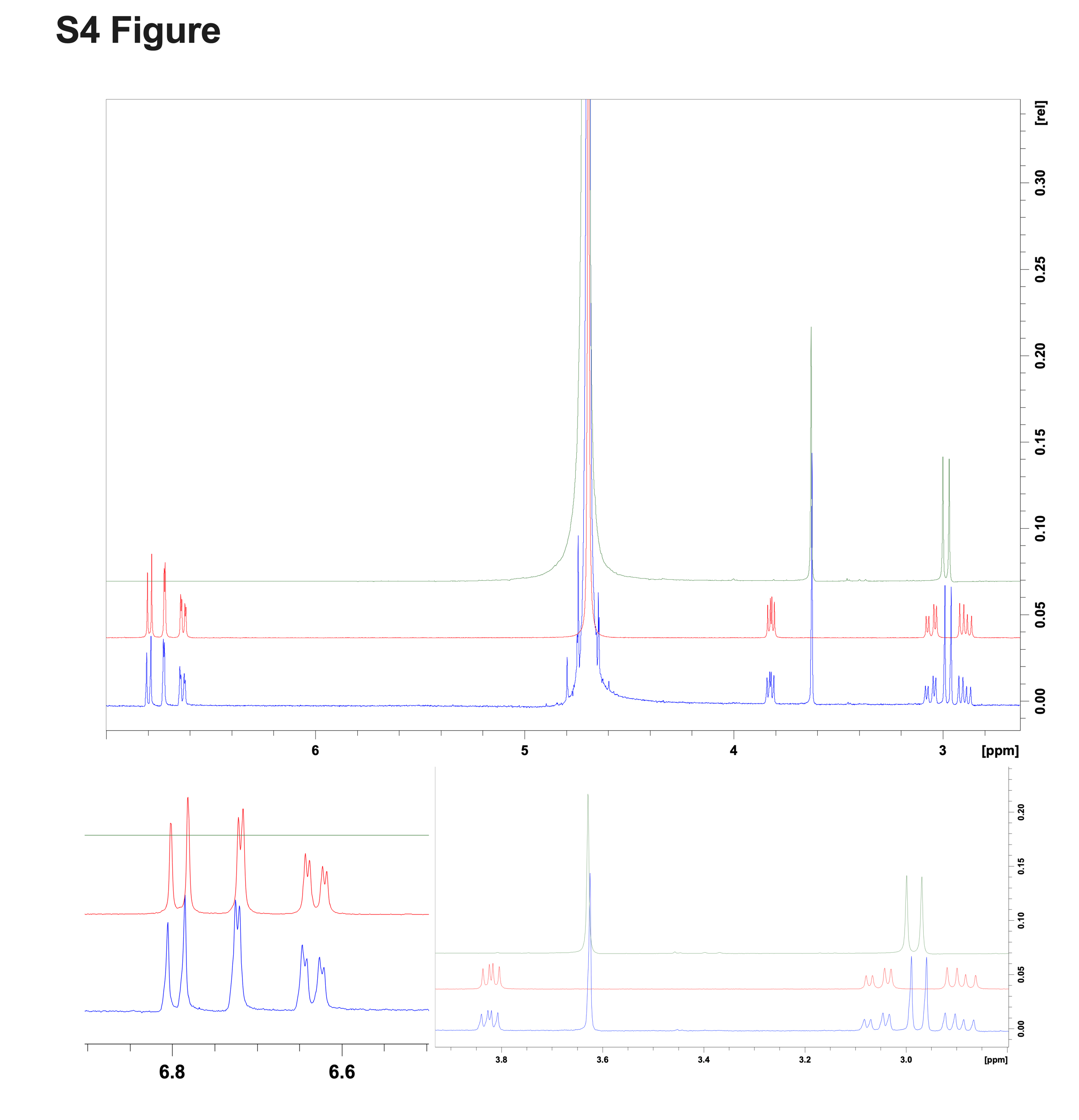

Supplement: S4 Fig — Representative 1H NMR spectra of 60 mM glyphosate solution in D2O (Green), 20 mM L-DOPA solution in D2O (Red), and 20 mM L-DOPA mixed with 60 mM glyphosate in D2O (Blue). There appears to be no shift in 1H peaks and no appearance of new peaks, which is indicative of no reaction occurring between the compounds. Data representative of findings from 3 independent replicates. For underlying data, please see Data Availability section and/or S1 Table. (TIFF) [file pbio.3001182.s004.tiff]

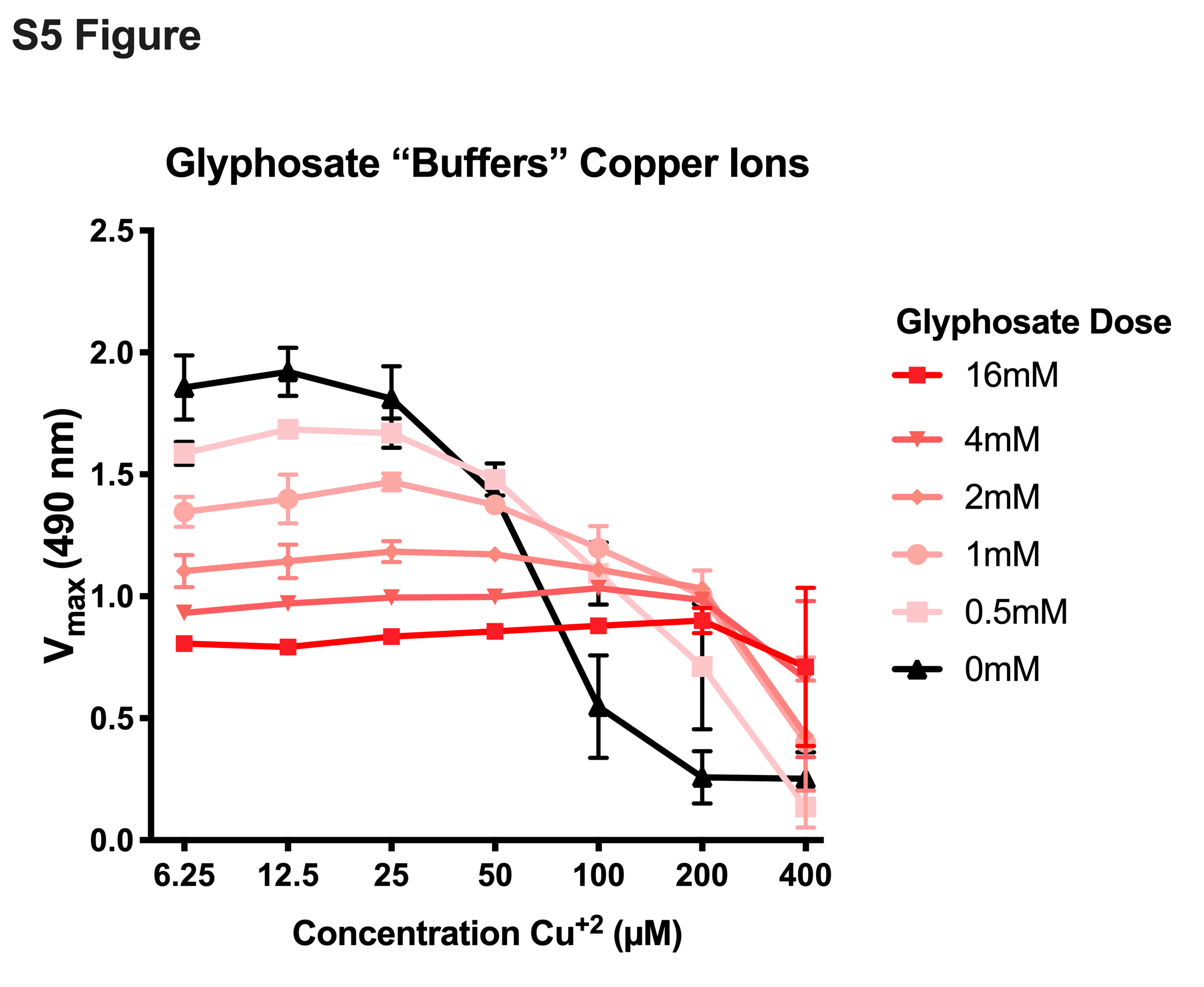

Supplement: S5 Fig — High doses (2–16 mM) of glyphosate prevent the enzymatic activity enhancing effects of lower copper concentration (6.25–25 μM), but high doses of glyphosate also prevent the enzyme inhibitory effects of high copper concentration (100–400 μM). Error bars represent ±SD. Data represent 2 independent replicates. For underlying data, please see Data Availability section and/or S1 Table. (TIFF) [file pbio.3001182.s005.tiff]

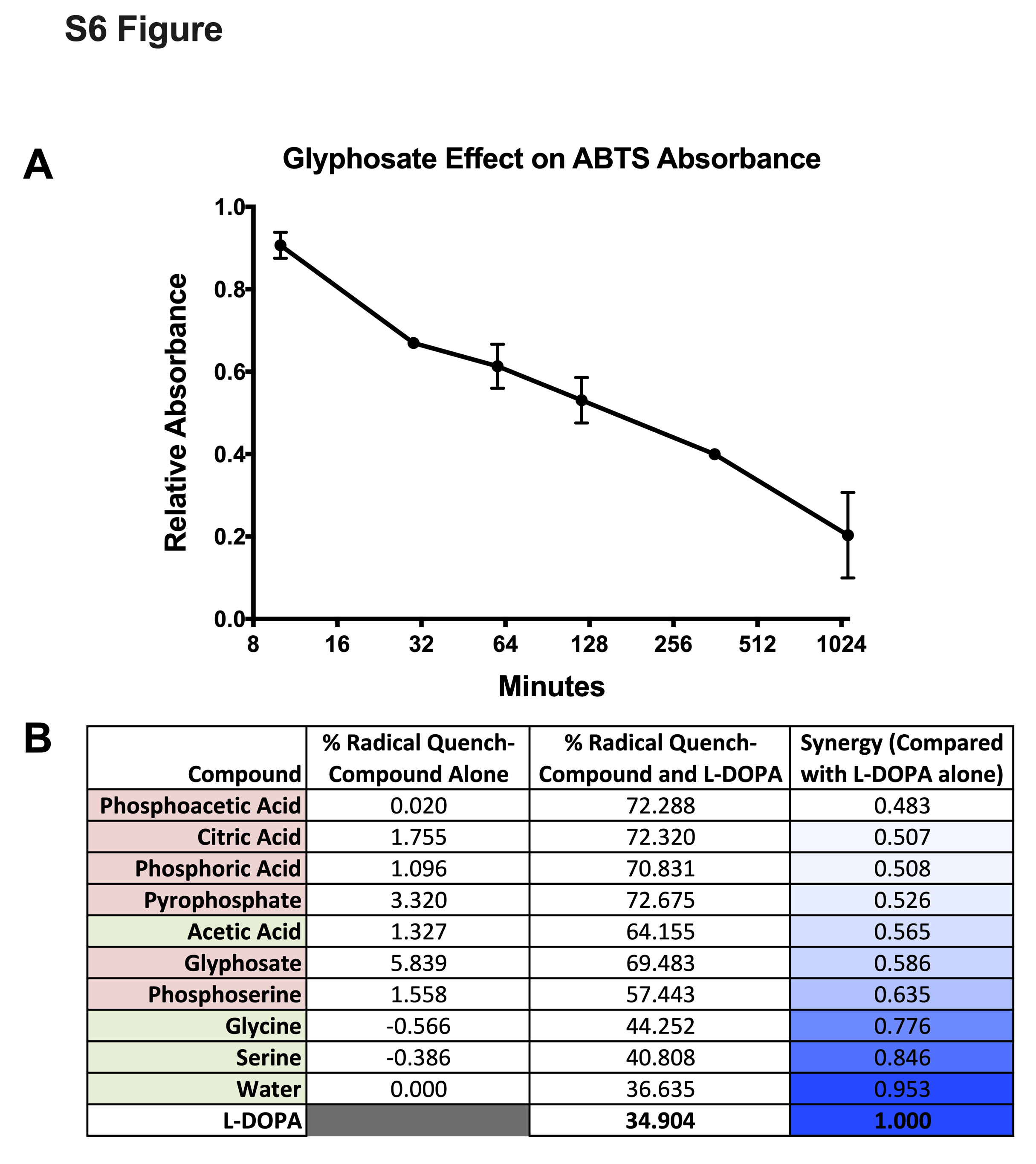

Supplement: S6 Fig — (A) Change in absorbance of ABTS solution at 734 nm over time for 33.33 mM glyphosate relative to the no glyphosate control. This indicates glyphosate quenches free radicals over an extended period of time. (B) Calculated antioxidant radical scavenging synergy between compounds tested and L-DOPA. Values represent the mean of at least 3 independent replicates. Error bars represent ±SD. For underlying data, please see Data Availability section and/or S1 Table. ABTS, 2,2′-azino-bis(3-ethylbenzothiazoline-6-sulfonic acid); L-DOPA, 3,4-dihydroxyphenylalanine. (TIFF) [file pbio.3001182.s006.tiff]

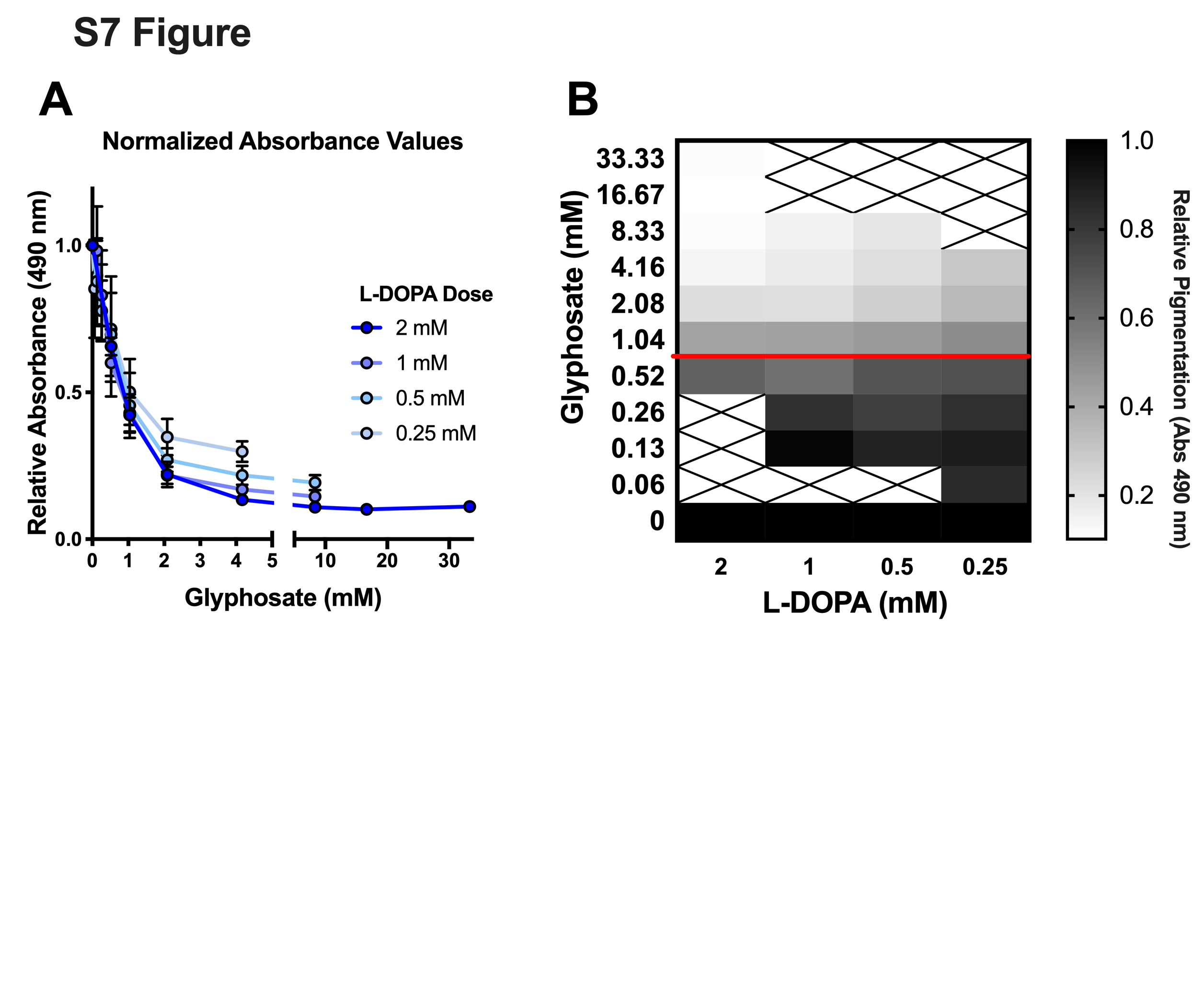

Supplement: S7 Fig — (A) Inhibitory concentrations of glyphosate are not affected by L-DOPA concentration. This indicates that glyphosate is not reacting proportionately with L-DOPA as measured by absorbance at 490 nm after 5 days of reaction, relative to the no glyphosate control and with background absorbance subtracted. (B) The IC50 of glyphosate remains constant at approximately 1 mM relative inhibition of melanin production appears dependent on glyphosate concentration alone and not on L-DOPA to glyphosate ratio. Data in panels (A) and (B) represent 2 alternative visualizations of the same experimental data. Error bars represent ±SD. Each experiment represents at least 3 independent replicates. Grayscale bars represent mean absorbance at 490 nm relative to no compound control. The darker colors correspond to increased pigment formation. Red line represents the approximate IC50. Crossed out boxes represent values with no data. For underlying data, please see Data Availability section and/or S1 Table. L-DOPA, 3,4-dihydroxyphenylalanine. (TIFF) [file pbio.3001182.s007.tiff]

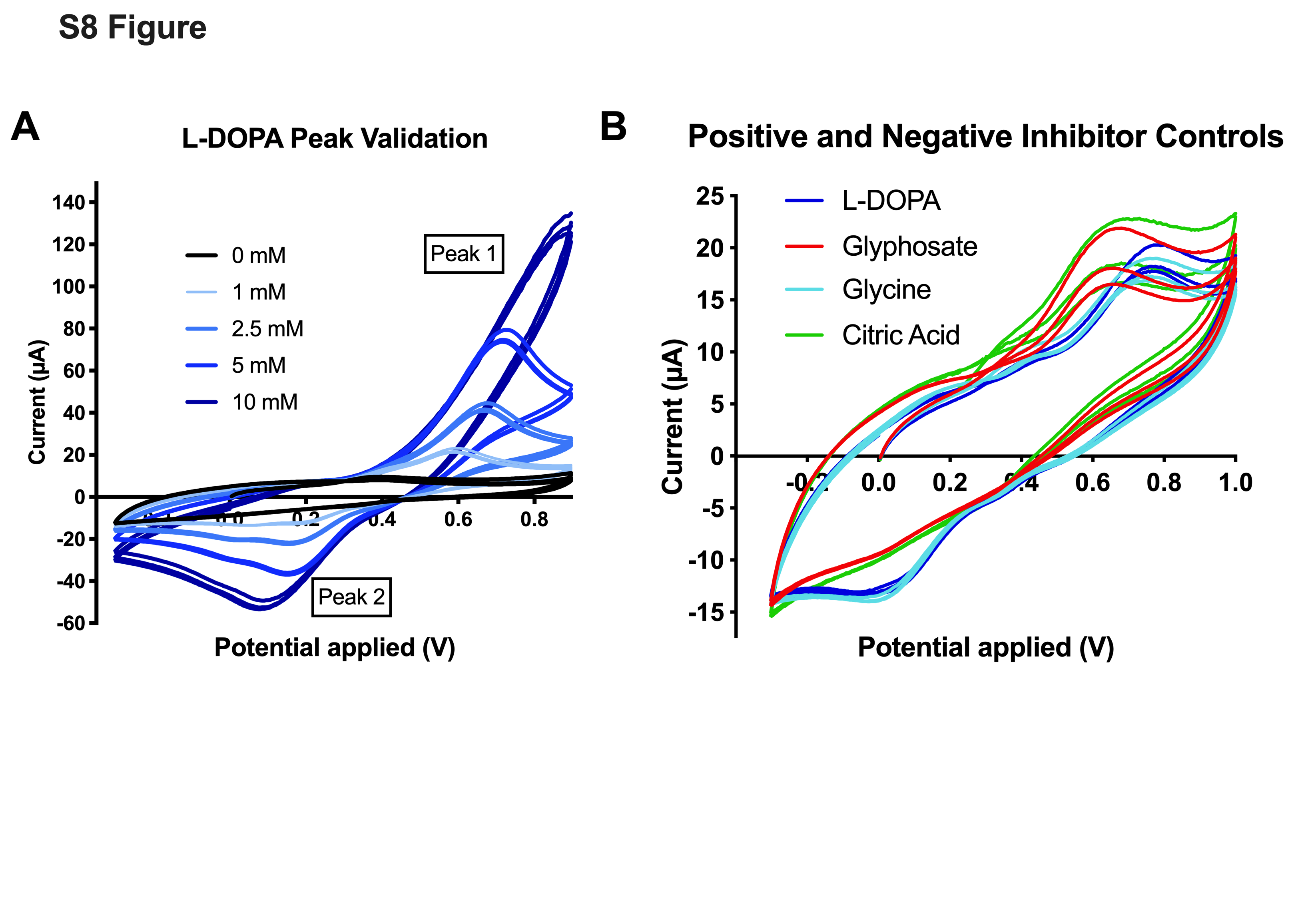

Supplement: S8 Fig — (A) Peak 1 was validated as the oxidation of L-DOPA, and Peak 2 was validated as the reduction peak of dopaquinone by correlating increased peak intensity with increasing concentration of L-DOPA under the same potentiostat parameters. (B) Glycine (16 mM)—a non-phosphate analog of glyphosate, a noninhibitor of melanization, and a nonantioxidant—does not alter the oxidation potential of L-DOPA. Conversely, citric acid (16 mM)—a known synergistic antioxidant and inhibitor of melanization—does alter the oxidation potential of L-DOPA in similar ways as glyphosate. The L-DOPA alone control and glyphosate voltammograms in panel (B) are the same as those found in Fig 7E. Each experiment represents at least 3 independent replicates, with 3 cycles per replicate. The tracings represent the mean value of the 3 replicates over the course of 3 cycles. For underlying data, please see Data Availability section and/or S1 Table. L-DOPA, 3,4-dihydroxyphenylalanine. (TIFF) [file pbio.3001182.s008.tiff]

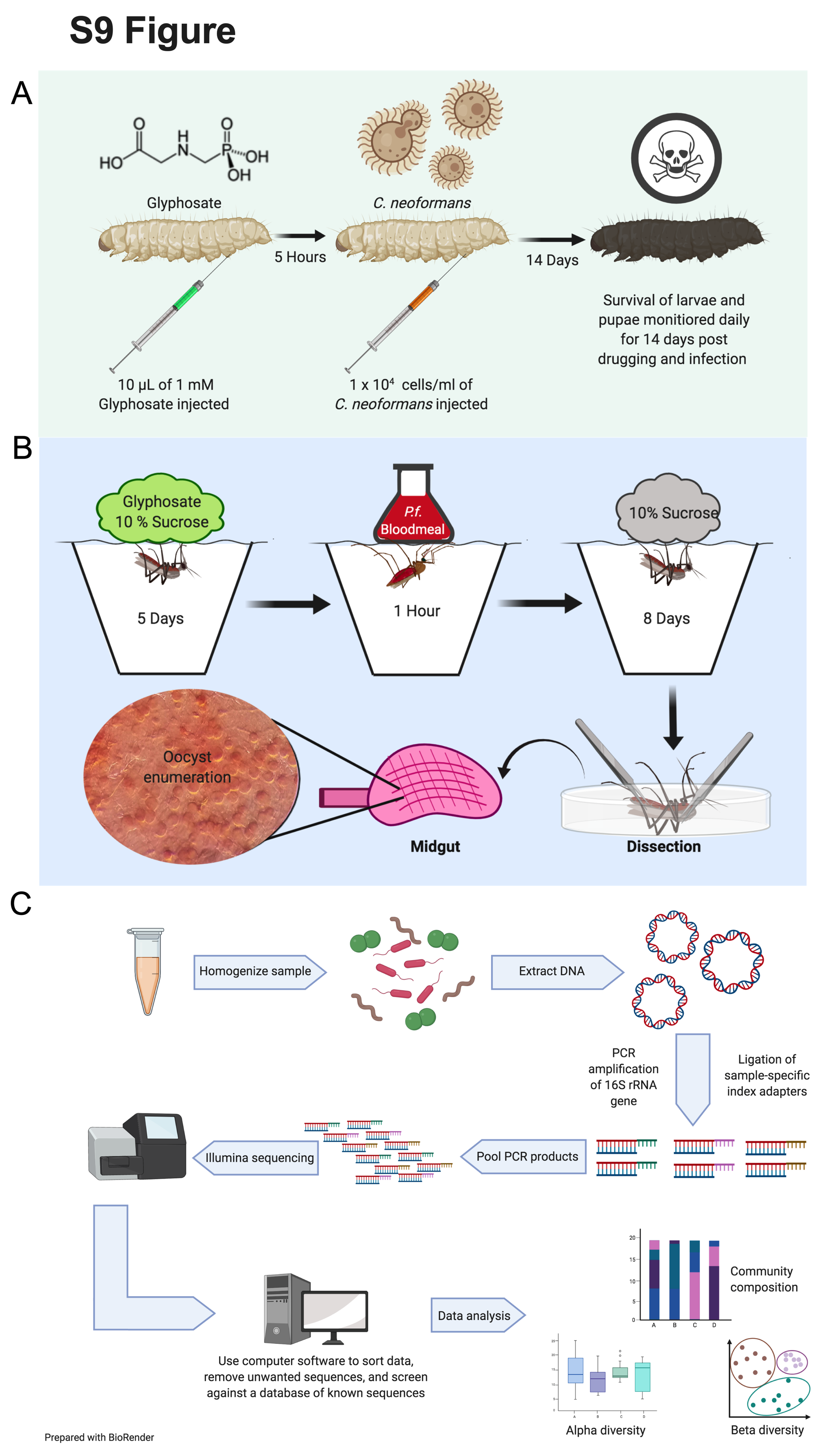

Supplement: S9 Fig — (A). During G. mellonella infection with C. neoformans, larvae were injected with 10 μl of 1 mM glyphosate, left to recover for 5 hours, and were subsequently infected with 104 cells/larvae of C. neoformans H99 strain. Survival was monitored for 14 days. (B) During A. gambiae infection with P. falciparum, mosquitoes were drugged with glyphosate-laced 10% sucrose solution for 5 days, then fed with a P. falciparum-infected blood meal, and fed 10% sucrose for 8 days. On day 8, mosquitoes were dissected, and the midguts were stained with mercurochrome to facilitate oocyst enumeration. (C) Glyphosate-drugged mosquitoes were dissected under sterile conditions, and 5 midguts were collected individually per condition. DNA was extracted from samples and bacterial 16S rRNA genes were amplified by PCR and sample-specific Illumina adapters were ligated to products. PCR products were pooled and sequenced on the Illumina MiSeq platform. Data were then analyzed using mothur to construct contigs, align reads, remove ambiguous bases and chimeric regions, align sequences to the Silva 16S V4 reference database, and cluster reads into 3% OTUs) Sequences from known contaminants were removed. Alpha and beta diversity measurements were performed using the Shannon diversity index and Bray–Curtis dissimilarity distance, respectively, and plotted using MicrobiomeAnalyst. Figures made with BioRender. OTU, operational taxonomic unit. (TIFF) [file pbio.3001182.s009.tiff]
