# Supplementary material for: Glyphosate inhibits melanization and increases susceptibility to infection in insects
Source: PLoS Biol. 2021 May 12;19(5):e3001182. doi: 10.1371/journal.pbio.3001182 (PMC8115815; doi:10.1371/journal.pbio.3001182)
Supplement: Attachment — Submitted filename: Reviewer Response Letter_ 11192020.pdf [file pbio.3001182.s012.pdf]

November 19, 2020

Dear Dr. Ines Alvarez-Garcia,

We appreciate the opportunity to revise and edit our previously submitted manuscript, "Glyphosate Inhibits Melanization and Increases Insect Susceptibility to Infection." Based on the reviewer comments, we have made substantial changes to the manuscript, as itemized and thoroughly detailed below.

One of the most substantial structural changes we have made was placing the insect experiments first, followed by the biochemical characterization data. Similarly, the abstract, introduction, and discussion have been edited to reflect this new order. Therefore the original Figures 1-4 and S1-5 have been changed to Figures 4-7 and S4-8 respectively. Original Figures 5-7 and S6-8 have been changed to Figures 1-3 and S1-3 respectively.

Broadly, we have updated the introduction to include more details regarding the positive effects of glyphosate in agriculture, its environmental fate, and its impact on non-target organisms. The revised introduction also includes more information on the melanin-based immune system of insects, and establishes the rationale for the experiments detailed in the Results section. Similarly, the discussion has been edited and restructured. The paragraphs of the Discussion have been reduced in length when possible. We have also included more information regarding the insect apocalypse, including discussion of academic skepticism regarding its breadth and severity.

Lastly, in response to some of the reviewer feedback, we have included new data for the *Galleria mellonella* and *Anopheles gambiae* findings. In *G. mellonella*, we have quantified the melanin-based immune response following *Cryptococcus neoformans* infection and glyphosate treatment. We have done so by enumerating and measuring the melanized nodules that form as an immune mechanism to eliminate the fungus. We have found that glyphosate reduces the magnitude and degree to which the *G. mellonella*'s melanin-based immune response encapsulates the fungus within melanized nodules (Figure 1D-G, S1G-I). We have also included new data showing that glyphosate does not impact pupation timing of the *G. mellonella* larvae (Figure S1F). We also include new data indicating that the glyphosate treatment of mosquitoes does not affect their cuticle pigmentation or their body size (Figure S2C,D). We have clarified the statistical tests performed and reanalyzed the statistics in Figures 1 and 2 to correct for multiple comparisons.

We would like to thank the Reviewer's for their helpful comments, suggestions, and feedback. We strongly believe that their help has improved the quality of our manuscript and the suggested experiments have strengthened our previous findings.

Sincerely,

Arturo Casadevall

Reviewer 1:

1. **Comment:** This is well-designed and timely study that extends previous findings showing that glyphosate inhibits melanization in fungi. In this study, Smith et al. demonstrate the molecular mechanisms by which glyph inhibits melanization and extend the effects to insects: they show that glyph also inhibits melanization in wax moth larvae and in malaria-transmitting mosquitoes, which leads to increased susceptibility to infection. I only have minor concerns regarding the study, which are described below.
   Response: We greatly appreciate the support and the immensely helpful feedback! Thank you.

2. **Comment:** Abstract, lines 30-32: It seems this sentence is missing a link after the word melanization, such as an "and".
   Response: Thank you for the edit suggestion! We have added**, "**and" added on Line 28after "melanization."

3. **Comment:** Lines 190, 507: Have you considered that glyph could be reversibly inhibiting tyrosinase, such as it does for the EPSP synthase, the main known target of glyph in plants and microbes? In this case, removing glyphosate or increasing the concentration of substrates would dislodge glyphosate from the enzyme, which would become active again.
   Response: Yes, this is something we have considered. To test this, we assessed Michaelis-Menten kinetics for the tyrosinase-DOPA reaction with different concentrations of glyphosate (Figure 3b originally, now 6b), resulting in a pattern consistent with non-competitive inhibition.
   We also tested the inhibition of the reaction with constant glyphosate and DOPA concentrations with different tyrosinase concentrations. For irreversible inhibition, we would expect similar slopes between the groups on a tyrosinase concentration vs activity plot - this would result in a consistent difference between the activities of the glyphosate-treated and untreated groups and a decreasing percent difference with increasing tyrosinase concentration. On the other hand, for reversible inhibition we would expect a lower slope for the glyphosate treated conditions, and this would result in a growing difference between the activities and a relatively constant percent inhibition with increasing tyrosinase concentrations. The data from this experiment is represented in Figure 3c (now 6c).
   Since the rest of our results indicate that the glyphosate-mediated inhibition is independent of enzyme activity, we had not emphasized this, as we believe the "non-competitive inhibition" is a result of disruption to the reaction's oxidative conditions not an irreversible effect on the enzyme. We have edited the manuscript to better describe the implications of Figure 3c in more detail/clarity, and we have visualized the experiment in Fig. 3c in a way that makes the findings clearer. Due to rearrangements of the manuscript, this figure is now Fig 6c.
4. **Comment:** Line 240: Figure citation is wrong
   Response: We have fixed the figure citation error.

5. **Comment:** Line 346: Figure 5a-b is not cited
   Response: Thank you for catching this, we have fixed it accordingly. 5a-b is now 1a-b and cited correctly.

6. **Comment:** Figure 6b: are there statistically significant differences in percent survival between groups? In other words, did you perform statistical analyses to corroborate what is said in lines 421-424?

Response: Thank you for pointing this out. We had not performed statistical analyses, but we have now done so using the Log-Rank Mantel-Cox analysis of the survival curves. We corrected for multiple comparisons using Bonferroni corrections. The change in survival for the 30 µM, 100 µM, 300 µM, and 10 mM all reach statistical significance, as now annotated in Figure 6b (now 2c) and described in the legends and manuscript where appropriate.

7. **Comment:** Figure 6c,d: it is not clear if statistical corrections were performed after multiple comparisons
   Response: Thank you for pointing this out. Originally, we had performed individual t-tests for each experimental concentration versus the control group. We did not perform statistical corrections for the multiple comparisons. For Figure 6c (now 2b), we have now performed the statistical analysis using a One-Way ANOVA with non-parametric ranks (Kruskal–Wallis Test), using Dunn's correction for multiple comparisons. We have updated Figure 6c (now 2b) to reflect the statistics from the new statistical analysis that is corrected for multiple comparisons. We have updated the text of the manuscript to make it clear that it is a trend of increased susceptibility and not one that is statistically significant. Although we have removed the panel that corresponded to Figure 6d, we have made sure than any statistical analyses have been corrected for multiple comparisons, either using Dunn's or Bonferroni methods

8. **Comment:** Lines 453: The use of CFU counts to investigate microbiome density/load is not appropriate, since not all microbes will grow under specific lab conditions. Ideally, quantitative PCR should be used to assess microbial loads
   Response: We appreciate the reviewer's concern. However, in this system we know that culturing captures the majority of gut associated microbes and as no impact was observed on the dominantly cultured microbes, we feel this is an adequate measure for our purposes. Moreover, in low density samples such as this, qPCR often gives artifacts and overestimates density, especially since most protocols utilize DNA as the template, which does not differentiate between living and dead bacteria in the gut, nor intracellular endosymbionts from bacteria within the gut. We performed the CFU experiment in accordance with previous literature (https://doi.org/10.1371/journal.ppat.1008453). Our culture-independent 16S rRNA sequencing was done to assess whether there were any changes in microbiome composition and not direct changes in abundance. To address the reviewer's comment, we have edited the text in the results to include a sentence about the shortcomings of using CFUs, and we have rephrased the text on CFU data to indicate that glyphosate did not "affect the density of culturable gut bacteria".

9. **Comment:** Lines 459, figure 7c,d: I could not find statistical support for the observed changes in alpha and beta diversity measures. Without statistically significant support, you cannot rule out that glyph affects A. gambiae microbiome in a dose-independent manner
   Response Thank you for noting this oversight. We now include the statistics for the alpha diversity measurement. There is no statistically significant difference between +/- GLYPH treated mosquitoes and no difference when broken down by concentration either. There is a statistically significant difference in the beta diversity metric. Taken together, these data suggest that while GLYPH does not alter the number of bacterial taxa present in each sample (alpha diversity), the community is perturbed by GLYPH and its composition is shifted in response (beta diversity). This is consistent with the taxonomic read-outs in Figure 7B (Now Figure 3B). The text has been updated to reflect these changes.

10. **Comment:** Why are the glyph-treated groups analyzed together in figure 7c,d, and not individually as in supplementary figure 8?
 Response: The PCA analysis of the Shannon Diversity indicated that the glyphosate-treated microbiota clustered together in a dose-independent fashion. Because of this, we opted to analyze whether there was an overall impact of glyphosate (+ vs -). We've added the stats on lack of a dose effect to the text and included the analysis with different doses to be transparent on the full data analysis as some might be interested in seeing the dose comparison.

Reviewer 2:

1. **Comment:** This manuscript describes two very different types of experimental studies, but the authors fail to provide a substantive basis for why they are presented together. For these reasons, I found the manuscript poorly organized and hard to follow, and rife with biased speculation about the consequences of GLYPH use.
 Response: Thank you for the constructive criticism. To ease understanding of this complex story, several sections of the manuscript were reorganized and edited. In addition, to further strengthen our findings we performed new experiments, as described below, to help develop a more cohesive research article.

2. **Comment:** First, in vitro studies demonstrated how glyphosate (GLYPH), a widely used herbicide, affects the melanization reaction of a fungal tyrosinase. The enzyme was incubated with GLYPH at different doses and conditions in the presence of different substrates and inhibitors. This work appears to be the first to investigate GLYPH inhibition of tyrosinase activity as no references for related studies were offered, so it could stand on its own in a separate manuscript submitted to a specialized journal.
 Response: Thank you for the recognition of our work, and the novel findings concerning the glyphosate's mechanism of melanin inhibition. While we do believe these findings could stand on their own in a specialized biochemical journal, we believe that this data have a greater impact presented it the context of our findings in insects.

3. **Comment:** The next set of studies examined the effects GLYPH on different aspects of melanogenesis in wax moth larvae and mosquito females. The authors appear to have little knowledge of related physiological processes in insects, fail to provide key background information about the insect models, and over interpret the importance of their results.
 Response: Additional descriptions in the introduction about the melanin-based immune system will help to clarify the relevant background and physiological processes. We believe that the new data may help bolster our claims.

4. **Comment:** The continued use and efficacy of GLYPH is highly controversial, and the authors should offer an unbiased over-view of the literature that encompasses the importance of GLYPH for inexpensive weed management to enable global food production along with possible environmental effects, including the so called 'insect apocalypse' that is only anecdotally reported for a few "instagram-able" insect groups and life stages, and worse still the bases for reports relying on meta-analyses,. Termites and ants largely inhabit soils and make up an estimated quarter to a third of the planet's animal mass - where's the data showing their decline or that of cockroaches or mosquitoes?
 Response: In the introduction and in the discussion, we have added a more comprehensive overview of GLYPH's fate in the environment, and we have included a sentence stating the

positive effects on global food production in recent years. We have also removed the sentences discussing the controversial/sensational claims of effects direct on human health.

Regarding the insect apocalypse, we have included sentences in the discussion that states the controversial aspects of the “insect apocalypse” hypothesis and have included several recent references detailing the controversy and data that are missing.

5. **Comment:** The author's premise for this work is that melanogenesis, one of several components of the insect immune response, may be affected by GLYPH persistence in the environment. No general description/comparison of cellular and humoral defenses, which would include melanogenesis, in insects was provided by the authors. My sense is that immune melanogenesis is a specialized pathogen/wound response in relatively few insects (e.g. melanization of parasitoid eggs in lepidopteran larvae), which the authors do not cover, and more properly, the emphasis should be effects of GLYPH on the phenol oxidase cascade as part of humoral immunity. The authors chose two very different insects and life stages for their studies, but offer no up-to-date reviews or references about this process specific to the insects (or related species).
Response: In our introduction section we have included more background information on melanization-based immune responses in insects, including references to roles that melanization plays in *Anopheles* adults and *Galleria* larvae. This process is conserved throughout all arthropods including insects, and are implicated in insect defense against a wide range of pathogens including bacteria, fungi, parasites, and even viruses. We also briefly describe the process of nodulation/encapsulation in which hemocytes aggregate around the pathogen and release immune factors and melanin capsule within the hemocyte aggregate.

6. **Comment:** Furthermore, the authors do not mention that given the above enzymatic characterization, GLYPH may similarly affect laccases and phenol oxidases/tyrosinases produced in other tissues that play perhaps even more important roles in insect development and reproduction. Such enzymes in the insect epidermis facilitate sclerotization and hardening of new cuticle after molting, and in the nervous system, dopamine produced by tyrosinase is an important neurotransmitter. Thus, the effects of GLYPH on wax moth survival after fungal infection primarily may be due to the failure to molt (altered dopamine neurotransmission?) or harden cuticle, given that larvae in the study presumably were fed and should have gone through one or molts within the 14 days they were monitored (Fig. 5 B). No mention of molting was made, and no direct evidence for melanization of the fungal pathogen in the larvae was given for the controls, so what link between GLYPH inhibition of melanogenesis and the multi-armed immunity in this insect is demonstrated by the data? None - just speculation - a more focused effort to assess the effects of GLYPH on other key immune pathways could possibly elevate the significance of this work.
Response: Thank you for the suggestions!

All of the *G. mellonella* experiments were performed in final instar larvae, so they do not molt post-infection, because of this they are also not consuming food as they prepare for pupation. As a result, we do not mention the molting process or diet in the results or methods. However, we have edited the manuscript to state “final instar” larvae in the text.

From our understanding, the *G. mellonella* larvae are cream-colored “soft-bodied” and also do not undergo significant sclerotization or cuticular melanization until they pupate (https://doi.org/10.1016/0045-6039(75)90046-9 and https://doi.org/10.1083/jcb.10.4.589), so we do not think the GLYPH would be increasing susceptibility through cuticular defects, moreover the timing of treatment and infection make that unlikely. Additionally, we have re-

analyzed data collected from the infection experiments (Figure 1c) in which larvae are treated once with GLYPH. During the experiments, we took note of pupation. We do not see significant changes in pupation rate between the PBS-treated and GLYPH-treated non-infected larvae at both room temperature and 30°C.

We appreciate the constructive criticism pointing out the direct link between GLYPH and reduced immune-mediated melanization. Previous studies have shown the *C. neoformans* induces nodule formation https://doi.org/10.4161/viru.29234), which is commonly seen with melanin encapsulation of fungus (https://www.ncbi.nlm.nih.gov/pmc/articles/PMC4194400). We performed additional experiments in which we drugged and infected the *G. mellonella* as described in the manuscript. After 24 hours we removed the hemolymph. We enumerated, measured, and characterized the melanized nodules within the hemolymph and find that there are smaller melanized puncta in the GLYPH-treated infected larvae compared to the PBS-treated infected larvae. PBS-treated uninfected larvae served as a control and had relatively few dark spots (nodules) in the hemolymph. We see that there are more fungi overall in the GLYPH-treated hemolymph, particularly non-melanin encapsulated fungi. We believe these data show that GLYPH directly reduces the melanin-based immune response and increases larval susceptibility to the fungus. We have included this data, including the melanized particle area, the number of particles per condition, the degree of melanin encapsulation, and representative images in Figures 1d-g and Supplementary Figure 1g-i.

We also have treated the *Anopheles* adults with 1 mM GLYPH for 5 days, as described in the *Plasmodium* infection protocol. We then evaluated cuticle pigmentation. We do not see any changes in the mean gray value of the cuticle following 5 days of GLYPH treatment (Supplementary Figure 2b). This was expected as the cuticular tanning of mosquitoes occurs during pupation and the first few days of adulthood prior to GLYPH treatment. In future projects, we would like to further pursue this angle of cuticular pigmentation defects, especially in the context of anti-desiccation properties and susceptibility to cuticle-penetrating pathogens.

7. **Comment:** As for the mosquitoes, perhaps GLYPH doses varied in their interference with neural transmission thus affecting survival after treatment (Fig. 6B).
   Response: It would be interesting in future studies to evaluate how glyphosate interferes with the mosquito's dopaminergic nervous system especially in the context of host-seeking and risk-avoidance behaviors, but this is beyond the scope of the current manuscript.

8. **Comment:** If there is no melanization of infective Plasmodium oocysts in GLYPH treated mosquitoes, then what is the link between GLYPH treatment, melanogenesis, and immunity that has a direct effect on Plasmodium infection and prevalence (Fig. 6D & D)?
   Response: There is no visible melanization in this model of *Plasmodium* infection. However, that does not rule interference with additional melanin-independent roles of phenoloxidases and catecholamines in immunity such as mediating parasite lysis through oxidative stress and toxic intermediates, or low-level deposition of melanin that cannot be seen visually. While we cannot say that the increased susceptibility of *A. gambiae* to *P. falciparum* is directly due to melanization, we think that the findings that glyphosate increases mosquito susceptibility to *P. falciparum* is noteworthy and important due to its implications to human health. See also response to point 10 below.

9. **Comment:** The fact that GLYPH is known to slow the growth of Plasmodium in cell culture may alone explain these results (Phillips, H. Could malaria be killed by a garden weedkiller? Nature (1998). https://doi.org/10.1038/news980702-2) - a point not addressed by the authors.
   Response: Our protocol involves pre-drugging the mosquitoes with glyphosate in sugar for 5 days , followed by 6 hour starvation, then administration of the infectious blood meal, so the glyphosate is not incubated directly with the parasite in culture to minimize this variable. Additionally, we do not believe that a slow growth of the parasite due to glyphosate would account for the increased parasite burden. Such an effect could be possible for the 10 mM group, however this result could be due to a hormesis-like response as well.

10. **Comment:** The inconsistent effects of GLYPH doses on the mosquito microbiota may also be due to direct inhibition of microbial pathways, as suggested by the authors, but that did not limit the speculation about melanogenesis, immunity, and the gut microbiota. Overall, the effects of GLYPH treatment on unrelated physiological processes in the two insects had little significance or coherence.
    Response: Previous studies have evaluated how the perturbed microbiome of insects affects their susceptibility to infection, including those showing that glyphosate disrupts the honeybee microbiome, which renders them more susceptible to infection. Given these previous studies, we felt it necessary to assess impacts on the microbiome to determine whether there was a similar effect in mosquitoes, as microbiome changes could partially explain the increased susceptibility we observe, as mentioned in the discussion. We do not link the perturbation of the microbiome to changes in the melanization immune response. These are two independent phenomena that are both caused by glyphosate and both have the potential to impact insect immunity and physiology (in accordance with previous studies). Furthermore, these experiments are significant to the insect microbiome field, as investigators work to see how this herbicide impacts different microbial communities.

11. **Comment:** Figures are not covered in the Results in an orderly manner (e.g. line 410, Fig. 6C is presented before 6B, and 6A is mentioned in the wax moth results section but not in the mosquito section). Several figure panels are not adequately described in the captions (e.g. Fig. 1A, and Fig. 2E what is shown in the inset?) and what exactly do the panels with black, gray, and white rectangles represent in Fig. 2D & E, Fig. 3D, Fig. 5C, and Sup. Fig. 4B, and where is the data analysis?
12. **Response:** Thank you for pointing out the disrupted order. The results section has been edited so that the figures are presented in the correct order for Figure 5 and 6 (now Figures 1 and 2). We have edited the figure legends of Figures 1C and 2E (now Figure 4D and 5E) to describe that the inset shows a representative photograph of the melanization inhibition with increasing concentration of glyphosate or other drug. They are images of the data represented in their respective figures. The grayscale heatmap figures in 2D, 2E, 3D, 5C, and Sup. 4B (now, 5D, 5E, 6D, 7C, Sup 1D, and Sup 7B respectively) represent the absorbance of the melanin pigment. The darker cell color on the heatmap corresponds to the higher relative absorbance and the darker color/more melanin produced. I have included, "Grayscale bars represent mean absorbance at 490 nm relative to no compound control. The darker colors correspond to increased pigment formation," in the captions of the figures that contain such heatmap.

13. **Comment:** In the caption for Fig. 6B, it is stated that "survival curves represent 120 animals across three biological replicates", so by my calculation that would be 12 mosquitoes for each of the 10 treatments, which for most survival studies is way too few. The numbers of Plasmodium

oocysts per midgut given for treated Anopheles females are exceptionally high in Fig. 6C compared to most studies and even in Sup. Figure 7, which is more commonly reported, but no explanation is offered for the count differences based on methodology or treatment between the mosquito cohorts or data sets.
Response: We apologize for the confusion. The 120 animals across 3 biological replicates is for **each** of the seven treatment groups. Therefore, each of the three biological replicates for each treatment group had 40 adult female mosquitoes, which comes out to a total of 896 mosquitoes used.

Regarding the oocyst burden, this is a typical median oocyst number in these strains of *Anopheles* and *P. falciparum* in lab settings (https://www.nature.com/articles/srep40520 , https://journals.plos.org/plospathogens/article?id=10.1371/journal.ppat.1000423, https://mbio.asm.org/content/8/5/e01631-17)

There is no difference in methodology between Figure 6C and Figure S7 (Now, Figure 2B and S2A, but some of the infections result in low infectivity with low parasite burden in control-infected mosquitoes which can make statistical comparisons difficult. Technical issues with the parasite culture may explained the low infectivity results, therefore advice we have received from other malaria researchers is to disregard these replicates, but we have chosen to include them as supplemental. These oocyst burdens are more in line with what is seen in nature, so we felt this data would be of interest to include, but not necessarily focus on

Reviewer 3: Sharon Pochron - this reviewer has waived anonymity

1. **Comment**: Overview: In a super interesting paper, the authors run a number of experiments that show that glyphosate exposure reduced wax moth larvae survival after infection, increased parasite burden in malaria-transmitting mosquitoes, and altered midgut microbiome composition in adult mosquitoes. The authors drew a line from their findings to the insect apocalypse, which makes the paper very powerful indeed.
Response: Thank you! We appreciate your enthusiastic support and the excitement about our results. We also appreciate the time you dedicated to the manuscript and the helpful suggestions, edits, and feedback!

2. **Comment**: Weaknesses: The manuscript is filled with grammatical errors, including run-on sentences. It is also poorly referenced, leaving out many important papers in the field, and using questionable references (e.g. meeting abstracts) for critical points, which is particularly annoying since solid references exist. I highly recommend the authors spend a day using Web of Science. Additionally, many of the key sections of the paper, even the title, are poorly argued.
Response: We appreciate your honest feedback, and the time you have taken to review our manuscript thoroughly. We believe the edits we have made have addressed these issues.

3. **Comment**: Strengths: The experiments, the results, and the implications stemming from the results are super important. The figures and illustrations are killer.
Response: We appreciate the recognition of our findings and of the illustrations!

4. **Comment:** Recommendation: Accept with major revisions, especially of the title, abstract, Introduction and Discussion.
Response: We appreciate the opportunity to revise the manuscript with the suggested edits.

5. **Comment**: Title: The long title is acceptable, but the short title makes no sense. The short title implies that the authors ran experiments on fungi, which they didn't. The abstract doesn't discuss fungi. Readers of the short title are left thinking, "Wait, what? Where's the fungi?"
   Response: By "fungi" we meant using fungal enzymes (i.e. the mushroom tyrosinase). However, since it may be misleading to readers, we have removed it and the short title is now, "Glyphosate Inhibits Melanization in Insects".

6. **Comment**: Abstract: Line 29, I'm not sure I'd use "environmental conditions." We think you really mean something like "exposure to ubiquitous contaminants."
   Response: We agree with your suggested phrasing since glyphosate is not a naturally occurring compound in the environment and is a contaminants. We have changed "environmental conditions" to "exposure to ubiquitous contaminants." (Line 17-18).

7. **Comment**: Lines 30-32: The authors state, "Here we elucidate the mechanism underlying glyphosate's inhibition of melanization demonstrate the herbicide's multifactorial effects on insects." The sentence makes no sense.
   Response: We edited this sentence during the abstract re-write and expanded it to: "Here, we demonstrate that glyphosate has deleterious effects on insect health in two evolutionary distant insect species, *Galleria mellonella* (Lepidoptera: Pyralidae) and *Anopheles gambiae* (Diptera: Culicidae), implying a broad effect in insects." (Lines 20-23), and , "We elucidated the mechanism by which glyphosate inhibits melanization, and showed that glyphosate acts as a synergistic antioxidant and disrupts the oxidation-reduction balance of melanization." (Lines 28-30).

8. **Comment**: Lines 30-33 should provide the set-up for the findings, which start on line 33. But I can't tell what the set-up is. The abstract should tell me why they ran the experiments that they ran. It doesn't.
   Response: We have edited the abstract and rearranged it to better reflect the paper structure, and to provide a smooth set-up into the rationale behind the experiments and our findings.

9. **Comment**: Line 33: While technically, glyphosate is a drug, I would use a more specific word here. Contaminant? Herbicide?
   Response: We have edited the abstract and refer to glyphosate as an herbicide and contaminant in the revised abstract and the following sections.

10. **Comment**: Line 36: so-called gets a hyphen.
    Response: This word has been deleted during general edits, as the direct reference to the "insect apocalypse" has been removed and replaced with "declines in insect populations." (Lines 32-33)

11. **Comment**: Introduction: So many run-on sentences. The first comma on line 46 should be a period, for example. Same for the comma on line 52.
    Response: We have split these run on sentences into two separate sentences each (Line 40 and 46).

12. **Comment**: Regarding the references in the first paragraph (lines 44 - 53), 3 of the 4 references cited are about insects-if you want to say something about ubiquity of tyrosinases, I suggest that you reference at least one more general paper about it. Also, if you care enough about

tyrosinases to put it into you keywords, you might want to state that they it is amongst the most widely employed enzymes as "green catalysts" for environmental applicability-and include a reference to that affect.
Response: Thank you for pointing this out. We have added more references that We believe fairly represent the assortment of tyrosinases across different kingdoms, including a book chapter that reviews general conserved mechanisms and structures (Line 43).

13. **Comment**: Line 61, the comma should be a semicolon.
Response: The sentence has been edited to. “Since melanization is an essential physiological process and effector of insect health, understanding how common environmental contaminants affect melanin production is important.” (Lines 61-63)

14. **Comment**: Line 63: You can't use a mouse paper to reference the statement "Glyphosate is a widespread herbicide found in the environment." There are a gazillion papers out there that show that glyphosate is everywhere. Start with Battaglin et al., 2014 and include Myers et al., 2016. Consider Bach et al., 2018 and Sihtmae at al., 2013. Laitinen et al. have a suite of papers on the topic. Additionally, glyphosate itself isn't the herbicide. It is the active ingredient in a family of herbicides. No one applies glyphosate alone to kill weeds. You make this point effectively in the next paragraph, so maybe consider revising your paragraph ordering?
Response: The mouse paper was in reference to the melanin-inhibiting properties of glyphosate in fungus and was not a reference to the widespread use of glyphosate (Lines 65-67). We appreciate the references and the suggested clarification on the nature of glyphosate use. We have included some of these references when discussing the wide use of glyphosate.

15. **Comment**: Still focusing on the paragraph that starts on line 63, I'm not sure what it's function is. What are you trying to communicate to the readers with it? I think you're trying to make the point that glyphosate inhibits melanization in fungus, but you start with the widespread contamination point and end with cosmetics. You need to revise your focus here.
Response: The purpose of the paragraph (starting Line 65) was to link, “why we care about melanin,” to, “why we care about glyphosate – particularly in the context of melanin,” but we understand how it appears scattered. We have moved parts of this paragraph to the discussion. We have kept glyphosate’s inhibition of fungal melanization as the beginning of the paragraph with the primary goal of transitioning to introducing glyphosate

16. **Comment**: Regarding your paragraph starting on line 71, you need to ask yourself what point you're trying to make here. It's highly unfocused. You start with the mechanism, wander into global contamination (again, and while missing major references on the topic; see above), wander in human cancers, and end on need to understand environmental impacts. That is a lot to ask of one paragraph.
Response: Thank you, we have restructured the introduction paragraphs in a way that we believe makes each paragraph more concise and focused on a single topic. For this paragraph, we have separated them into three separate paragraphs with the focus of 1) What is glyphosate? (Starting Line 65) 2) How much glyphosate is found in the environment and where? (Starting Line 75), and 3) What is the fate of glyphosate? (Starting line 84).

17. **Comment**: Regarding the paragraph that starts on line 84, you are missing critical references. It should include Battaglin et al., 2014 and Gill et al. 2018. Gill et al., 2018, reviews the impact of glyphosate on all animals. Your review of the impact of glyphosate on other insects is

depauperate. You should include: (Baglan et al., 2018) (mosquitos); (Tahir et al., 2019) (spiders); (Farina et al., 2019; Tomé et al., 2020) (bees).
Response: Thank you for these references! They have taught us a tremendous amount about glyphosate and its impact. We have tried to include these references in a paragraph with the main topic of “effects of glyphosate on animals” (starting line 102), outside of the paragraph dealing with microbes and insect microbiome perturbations

18. **Comment**: Also, like your earlier paragraphs, the paragraph that starts on line 84 is unfocused, in addition to being poorly referenced. If you're going to discuss the half life of of glyphosate, you should be using more recent review papers such as (Singh et al. 2020) and (Van Bruggen et al. 2018). If you're going to discuss how long it can remain in the top layer of soil, you should be citing all of the Laitinen papers. There is a rich body of literature discussing the impact of glyphosate on soil microbes and fungi. Look at Pochron et al. 2020 for a review of that.
Response: Thank you again for the references, and we have read them and found them particularly helpful to our understanding of glyphosate’s “lifecycle” in the environment. We tried to include the references as suggested.

19. **Comment**: But again, pick the point you want to make and make it in one paragraph. This paragraph wanders from run-off, to environmental concentrations, to the impact on soil microbes and its subsequent impact on plants, back to environmental concentrations, to the impact on algae, to the impact on bee microbiomes and fly microbiomes. No reader can make sense of the story you're trying to tell.
Response: We have split these paragraphs up, as previously mentioned to help them flow better. The new structure is as follows: 1) how much glyphosate is found in the environment and where? Line 75, 2) what is the fate of glyphosate in the environment? Line 84, which is where the bulk of the discussion of glyphosate’s interaction with microbial communities (in soil and in insects) is discussed, and 3) what does glyphosate do to animals beyond disturbing microbial communities? Line 102.

20. **Comment**: In your final Introduction paragraph (line 96), you introduce the reader to a mushroom tyrosinase model with no explanation. You toss in a bunch of scientific names, without the common names, and you sketch out your experiment. At this point in the Intro, I should be able to determine how and why your experiment is important. It should tell me why you, the authors, think that glyphosate impacts melanin production. You don't. I have no idea why you're running these experiments.
Response: We have rearranged the paragraph to introduce the insect information first followed by the mushroom tyrosinase (starting Line 114). A better explanation of the rationale for the mushroom tyrosinase experiments is provided. Scientific names with the organisms’ relevance have also been explained (i.e., “pathogenic fungus *C. neoformans*,” “*Galleria mellonella* – a species of wax moth larvae,” and, “*Anopheles gambiae* – a mosquito vector for human malaria.”)

21. **Comment**: Lastly, your Introduction and Discussion should bear some resemblance to each other. Your Discussion opens with AMPA, but AMPA is not discussed in your Intro.
Response: Additional background about AMPA (its formation, stability, localization, and effects on insects) has been included in the introduction.

22. **Comment**: For each of the outcome variables you measure in your Results (and you have many! All interesting!), you need to touch on those in your Intro. What impact do you expect to see in gut microbiomes, body mass, survivorship with and without infection, oocysts per midgut (or other measures of immune system, and all those things with mushrooms when you expose your models to glyphosate and AMPA? Your Intro should be a review of THAT literature. Since you have the insect apocalypse in your abstract, that should be in your Intro too.
Response: We appreciate the suggestion. The closing paragraph of the introduction has been expanded to provide a rationale for the experiments we have performed, the models we have used, and the output of these experiments. we could not find an appropriate section in the introduction to include much information about the insect apocalypse. We have removed the phrase "insect apocalypse" from the abstract and replaced it with "contribution to insect declines, and we discuss the insect apocalypse in a more substance in the revised discussion

23. **Comment**: The Introduction needs to be completely revised.
Response: Considering the suggested changes, the introduction section has been restructured, additional information and citations were included.

24. **Comment**: Given that PLOS Biology aims its papers are a more general readership than other more specialized journals, I recommend that you use fewer acronyms. PO, DQ, GLYPH, MBTH. Just use the real words. The readers don't have to translate them continually and that improves readership.
Response: Thank you for the suggestions. we will change "GLYPH" to "glyphosate," "PO" to "phenoloxidase," "DQ" to "dopaquinone," and "DC" to "dopachrome". We will keep the MBTH abbreviation because we think its full name might be too cumbersome.

25. **Comment**: I think that experiments 1-4 used some sort of mushroom model and that the remaining experiments use insect models. The results section might benefit from giving some sort of overview as to what experiments were run and what kind of model was used. (The Intro should cover that too, as well as tell us why.)
Response: We have edited the results section of the manuscript. We think the new section entitled "Glyphosate Inhibits Production of Dopaquinone, Dopachrome, and Melanin," gives an overview of the tyrosinase reaction steps and its similarities to insect phenoloxidases, especially in conjunction with the revised introduction sections.

26. **Comment**: Much of the paragraph starting on line 105 doesn't belong in Results. It belongs both in the Introduction and the Materials and Methods. Results sections should include only results.
Response: We have consolidated the section and combined it with the subsequent "Dopachrome and Melanin" section. Some of the paragraph remains to help transition from the insect to tyrosinase data, but most of the section was moved to methods or deleted it entirely.

27. **Comment**: Line 108, you have a sentence that begins with "First" but there is no second.
Response: Thank you for pointing this out. "First," changed to, "The first step of the reaction involves…" (Line 243).

28. **Comment**: Actual results start on line 118. I am not an expert on methods of evaluating melanization. I can clearly see the dose dependent inhibition in Figure 1A, but I cannot understand this experiment given the Results and Figure 1A. A quick word-based sketch of what this experiment was supposed to do and show would benefit your readers. Something was

tyrosinase mediated and something else was auto-mediated, but I can't figure out your model. You should be able to communicate this model to other glyphosate specialists who are unfamiliar with mushrooms and melanization. Also, on line 120, you state that it appears that the inhibition in the reaction is due to inhibited background auto-oxidation. Please explain how you come to that conclusion. Why did you run the experiment with and without tyrosinase?
Response: The final section of the introduction and the opening paragraph for this section in the results has been restructured and edited to facilitate clarity and understanding. This figure was moved to Figure 4. We have also included the melanization pathway (Mason-Raper pathway) in Figure 4A.

Since L-DOPA is spontaneously oxidized (auto-oxidized) into DQ as well as by tyrosinase, we performed the MBTH experiments to look at DQ formation during enzyme-mediated and autooxidation-mediated production of DQ during inhibition with glyphosate. We see that the slope of inhibition of DQ formation for the autooxidation and the tyrosinase reactions are similar. This leads us to believe that if the "background" autoxidation data is subtracted from the tyrosinase data, the tyrosinase reaction DQ levels would be constant. This indicates to us that the inhibition of DQ is primarily rooted in preventing the oxidation of L-DOPA independent of the tyrosinase enzyme. We have updated the manuscript to include this explanation (Lines 248-251).

29. **Comment**: Section starting line 124: Clarifying as described in the paragraph immediately preceding this paragraph will benefit readers of your second experiment. Why do we care about the rate and level of DC produced? What does it tell us?
Response: DC is a straightforward melanin intermediate to measure between the formation of DQ and melanin. The rate and level of DC produced tell us additional information about what melanin intermediates are formed during glyphosate inhibition. Evaluating both DQ and DC provides information about the radical-mediated redox exchange (which is sandwiched between the DQ and DC steps of melanization). DC is also a very commonly used intermediate to measure tyrosinase/phenoloxidase activity since it has a distinct absorption peak and does not require derivatization or reaction with another compounds (i.e. MBTH). This is now explained in Lines 255-256).

30. **Comment**: The experiment describe starting line 150 is really cool! The authors looked at non-phosphate analogs of glyphosate on DQ (melanin) production and compared them to phosphate-based analogs and glyphosate itself. Why did you home in on P? That should be in your introduction. Make non-experts care about this experiment. Make it clear that measuring DQ is a measure of melanin. Did you still use the mushroom model? Even adding a mushroom icon to your figures might help communicate your model more effectively. In 2F, consider putting the names of the compounds under their structures. Figuring out the name of each compound and finding it on the curves was nearly impossible.
Response: We honed in on the phosphate group by testing glycine compared to glyphosate, and serine compared to phosphoserine. We found that the phosphate compounds had inhibitory properties while the non-phosphate compounds did not. we have edited the manuscript to make it clear that we are measuring DQ, DC, and melanin, and that we are are using the *in vitro* mushroom tyrosinase model for these experiments (Fig 5a-c). Additionally, we have put the names of the compounds under their structures to facilitate finding the compounds and associating structure, name, and inhibitory properties.

31. **Comment**: Line 163, you state, "GLYPH and similar compounds inhibit melanin in a non-enzymatic fashion." If I understand the experiment correctly, you selected phosphate-based compounds and non-phosphate-based compounds to determine which part of the glyphosate molecule drove the effect. (If we didn't understand the experiment, please clarify it.) In your results, you need to describe the bimodal distribution-state that it's based on P, which is pretty cool.
Response: The experiments were understood by reviewers. However, "…similar phosphate-containing compounds…" (line 282) was added to ensure clarity. In lines 283-284, "These data suggest that the phosphate functional groups of these compounds may be responsible for the melanin-inhibitory properties," was included to emphasize the role that we believe the phosphate groups are playing in the inhibition.

32. **Comment**: Line 214. You need one more sentence that interprets what the result means.
Response: we agree. Sentence added that states "This result indicates that GLYPH's ability to chelate copper ions could have a protective effect in high copper environments, which would otherwise lead to negative effects on enzymatic activity and other biological processes." (Lines 320-322).

33. **Comment**: Line 339. Consider leading your results section with the insect models and then moving to the mushroom models.
Response: Thank you for your recommendation. We have rearranged the results section to develop the insect models first, followed by the mushroom tyrosinase.

34. **Comment**: Line 357: If you're going to use AMPA in your experiments (and I agree you should!), you need to discuss it in your Introduction.
Response: We agree with your suggestion. We have added sentences in the introduction and the discussion that reference AMPA's presence and persistence in the environment and its (lack of) effect on honeybee microbiota.

35. **Comment**: Line 358. You call out Fig 5 for the first time, after Fig 6. Noooo.
Response: We have edited the results to include the Figure 6a (now 2a) with the rest of the Figure 6 (Figure 2) data in correct order.

36. **Comment**: Lines 353-354 and 357-358 are highlights of this paper. They're the reason we agreed to review this manuscript, and they're the reason people will want to read the paper. You need to use all of your skills as writers to make your readers understand why those are interesting questions and why it is amazingly cool that you found your answers**.** Your Intro should be written so as to make me want to know what the impact of glyphosate and AMPA are on insect melanin production.
Response: We think that the revised introduction, results, and discussion sections do an overall better job at showing why the effect of glyphosate and AMPA on melanization is impactful and interesting findings. Again, we are thankful for your enthusiasm and support.

37. **Comment**: In Figure 6, you misspelled Fisher.
Response: Thank you for pointing this out. The panel corresponding to this part of the legend been removed, but "Fisher" in Supplementary Figure 2b has been corrected.

38. **Comment**: Lines 419-424. You need to get into the **hormesis** literature. My apologies. It isn't fun.
    Response: Thank you for the suggestion. It was very interesting to read overall reviews about hormesis and potential mechanisms into why a lot of compounds have a hormesis-like effect. we think the information will be helpful in interpreting future results, as well as planning for experiments in which there should be a dose-dependent exposure to a certain compound. we have included mentions of hormesis in the copper toxicity section of the results as well as the *A. gambiae* survival sections. we do not go into detail concerning hormesis, but we think it was helpful to broaden our understanding.

39. **Comment**: Your experiments regarding insects are easier to understand and interpret than your results with the fungi. You need to set up the fungi experiments better. Something like, once we found these cool results with bugs, we wanted to find the mechanisms so we did this cool thing with a mushroom model. Because of this, please consider leading your results with the insect experiments and leading people to care about the mushroom results. It'll make for a better read. Leading with the mechanism is slightly painful to read.
    Response: Thank you for the suggestion. We have re-arranged the results accordingly to include the insect data first.

40. **Comment**: How close are the glyphosate doses you used in your insect experiments to those that they may encounter in nature? I mean when you injected the glyphosate.
    Response: For the *Galleria* experiments, we chose the concentration based on the fungistatic effect of glyphosate on *C. neoformans* as shown in previous literature (Nosanchuk et al. 2001). We did not want to inhibit fungal growth and interfere with its ability to infect the larvae. From each larvae, we were usually able to extract ~ 50 µl of hemolymph, so in the scenario in which the glyphosate only stays in the hemolymph, the 10 µl of 1 mM glyphosate will get diluted to about 250 µM, which is the highest concentration that *C. neoformans* still grows normally, and a concentration of glyphosate reasonably found in nature. In the other scenario in which glyphosate is evenly distributed throughout the larvae's tissues, the final concentration of the herbicide would be about 50 µM (the average larvae volume used would be about 200 µl based on water displacement tests).

41. **Comment**: Line 485: what is it?
    Response: The discussion has been restructured, so this sentence has been deleted.

42. **Comment**: You have a paragraph on page 21 that covers almost an entire page. No one can read that. In fact, I recommend that you do through your Discussion and make sure all of your paragraph are no more than 1/3rd of a page. In their current form, your paragraphs are driving away your readers, which will not improve the number of people who reference this awesome paper.
    Response: Thank you for the suggestion. We have tried to reduce the size of many, if not all the paragraphs of the discussion, as well as throughout the manuscript. There are occasional paragraphs that take half a page in order to keep the same thoughts together in one paragraph rather than divide them.

43. **Comment**: Your opening sentence of that paragraph (line 520) doesn't make as much sense as one might hope. Your phrase, "which are necessary." modifies solution but I think you want it to

modify free radicals but maybe you want it to modify inhibitors. Line 524, the comma should be a period.
Response: Thank you for the suggestion, and we think the new change has made the sentence more clear. The opening sentence has been changed to, "We examined the ability of glyphosate and the other compounds to quench free radicals, which are necessary to the melanization process." (Lines 499-500). The sentence corresponding to Line 524's comma (now lines 502-504) has been restructured.

44. **Comment**: Lines 558-560. I object to using the same suite of references to make the point that glyphosate changes ecosystems by disturbing microbial populations AND inducing oxidative stress. Those should be two sets of references. ALSO, there are many, many papers that show that glyphosate has no impact or only a fleeting impact on ecosystems. (Most of those paper measure microbial biomass rather than community structure, but still, you should acknowledge that.) See for instance: Gornish, E.S., Franklin, K., Rowe, J. and Barberán, A., 2020. Buffelgrass invasion and glyphosate effects on desert soil microbiome communities. Biological Invasions, pp.1-11. Also, read the Discussion of Pochron et al. 2020 for the nuances involved in predicting the soil microbial response to glyphosate and Roundup exposure. The jury is still out on how bad glyphosate is for microbial systems.
Response: Thank you for the list of suggested reference. The references we had previously used were intended to just discuss the oxidative stress induction by glyphosate. We have removed the microbial component of the sentence and focused on the oxidative stress. We have included a discussion of the (potential) effects on microbes and microbial communities in the revised introduction of the manuscript using some of these references and previously suggested ones.

45. **Comment**: Lines 580-581: Body weight does not always respond to exposure to contamination by decreasing (e.g. Pochron et al. 2018; 2019); in fact, sometimes organisms increase their body size in response to exposure to various contaminants, leading to an entire body of literature dedicated to hormesis, defined as a favorable biological response to exposure to toxins or other stressors (Agathokleous et al. 2019; Docea et al. 2019; Wang et al. 2018). Hormesis is most likely to occur at low dose exposures, and it doesn't involve just glyphosate. The literature includes examples involving heavy metals and parasites. You are not alone in your vexing result regarding body weight.
Response: Thank you for the information regarding hormesis and body mass changes. This information has already proven useful beyond the scope of this project, as we have planned protocols for other experiments to take the possibility/likelihood of hormesis and a biphasic response under consideration.
We have not directly looked at how the *Galleria* and *Anopheles* body weights are affected by glyphosate drugging, however in response, we have measured the wing lengths of glyphosate-treated and control-treated *A. gambiae* adults following protocols performed in the *P. falciparum* susceptibility experiments. Wing length is used as a proxy for body mass (https://doi.org/10.1093/jmedent/33.2.261) in mosquitoes. We do not find a significant difference in wing length between the glyphosate and control groups, and we have included this in Supplemental Fig 2C. It would be interesting to investigate in future projects looking at glyphosate exposure throughout the life cycle and across several developmental stages (i.e. exposure of larvae and pupae to the drug and seeing how adult body weight is impacted).

46. **Comment**: Line 564: This paragraph is too long.

Response: We agree, thanks. Throughout the manuscript, length of paragraphs, including this one, have been split and edited down into smaller sections when possible.

47. **Comment**: Line 608: You spend a lot of time talking about concentrations in run off. You've missed the literature showing that glyphosate and AMPA occur in our rain, soil, sediments. It would be nice if you could tie that universal contamination in to the ecosystem services delivered by insects. I know that no one will mourn the death of mosquitos, but they are good models for more beneficial insects. You can draw that line. I know nothing about your moth, but if its larva generally lives in the soil, then there is a whole body of literature about the ecosystem services delivered by soil-dwelling insects. People are going to read this paper because of the insect apocalypse angle. You've buried that lead in your text. I recommend that you bring it out front and center and make the mechanism the part of your manuscript that gives teeth to your experiments showing that glyphosate is bad for insects. Right now, this is paper is no fun to read. You can make it a lot better by focusing your Introduction and Discussion.
    Response: Thank you for that suggestion. We have included additional information regarding where Glyphosate and AMPA are found. We have rearranged the results to focus on the insect data first followed by the mechanism data as you have suggested. We think with the additional edits, it is clearer that these findings could apply to additional insects in ecosystems. The *Galleria* larvae live in honeycombs and eat the wax. While they do not typically live in soil, although other caterpillars do.
