# Supplementary material for: Glyphosate inhibits melanization and increases susceptibility to infection in insects
Source: PLoS Biol. 2021 May 12;19(5):e3001182. doi: 10.1371/journal.pbio.3001182 (PMC8115815; doi:10.1371/journal.pbio.3001182)
Supplement: Attachment — Submitted filename: PLOS Bio review Smith et al.docx [file pbio.3001182.s011.docx]

Review of: Glyphosate Inhibits Melanization and Increases Insect Susceptibility to Infection (Glyphosate Inhibits Melanization in Insects and Fungi).

**Overview**: In a super interesting paper, the authors run a number of experiments that show that glyphosate exposure reduced wax moth larvae survival after infection, increased parasite burden in malaria-transmitting mosquitoes, and altered midgut microbiome composition in adult mosquitoes. The authors drew a line from their findings to the insect apocalypse, which makes the paper very powerful indeed.

**Weaknesses**: The manuscript is filled with grammatical errors, including run-on sentences. It is also poorly referenced, leaving out many important papers in the field, and using questionable references (e.g. meeting abstracts) for critical points, which is particularly annoying since solid references exist. I highly recommend the authors spend a day using Web of Science. Additionally, many of the key sections of the paper, even the title, are poorly argued.

**Strengths**: The experiments, the results, and the implications stemming from the results are super important. The figures and illustrations are killer.

**Recommendation**: Accept with major revisions, especially of the title, abstract, Introduction and Discussion.

**Title**: The long title is acceptable, but the short title makes no sense. The short title implies that the authors ran experiments on fungi, which they didn’t. The abstract doesn’t discuss fungi. Readers of the short title are left thinking, “Wait, what? Where’s the fungi?”

**Abstract**: Line 29, I’m not sure I’d use “environmental conditions.” I think you really mean something like “exposure to ubiquitous contaminants.”

Lines 30-32: The authors state, “Here we elucidate the mechanism underlying glyphosate’s inhibition of melanization demonstrate the herbicide’s multifactorial effects on insects.” The sentence makes no sense.

Lines 30-33 should provide the set-up for the findings, which start on line 33. But I can’t tell what the set-up is. The abstract should tell me why they ran the experiments that they ran. It doesn’t.

Line 33: While technically, glyphosate is a *drug*, I would use a more specific word here. *Contaminant*? *Herbicide*?

Line 36: so-called gets a hyphen.

**Introduction:** So many run-on sentences. The first comma on line 46 should be a period, for example. Same for the comma on line 52. Regarding the references in the first paragraph (lines 44 – 53), 3 of the 4 references cited are about insects—if you want to say something about ubiquity of tyrosinases, I suggest that you reference at least one more general paper about it. Also, if you care enough about tyrosinases to put it into you keywords, you might want to state that they it is amongst the most widely employed enzymes as “green catalysts” for environmental applicability—and include a reference to that affect.

Line 61, the comma should be a semicolon.

Line 63: You can’t use a mouse paper to reference the statement “Glyphosate is a widespread herbicide found in the environment.” There are a gazillion papers out there that show that glyphosate is everywhere. Start with Battaglin et al., 2014 and include Myers et al., 2016. Consider Bach et al., 2018 and Sihtmae at al., 2013. Laitinen et al. have a suite of papers on the topic. Additionally, glyphosate itself isn’t the herbicide. It is the active ingredient in a family of herbicides. No one applies glyphosate alone to kill weeds. You make this point effectively in the next paragraph, so maybe consider revising your paragraph ordering?

Still focusing on the paragraph that starts on line 63, I’m not sure what it’s function is. What are you trying to communicate to the readers with it? I think you’re trying to make the point that glyphosate inhibits melanization in fungus, but you start with the widespread contamination point and end with cosmetics. You need to revise your focus here.

Regarding your paragraph starting on line 71, you need to ask yourself what point you’re trying to make here. It’s highly unfocused. You start with the mechanism, wander into global contamination (again, and while missing major references on the topic; see above), wander in human cancers, and end on need to understand environmental impacts. That is a lot to ask of one paragraph.

Regarding the paragraph that starts on line 84, you are missing critical references. It should include Battaglin et al., 2014 and Gill et al. 2018. Gill et al., 2018, reviews the impact of glyphosate on all animals. Your review of the impact of glyphosate on other insects is depauperate. You should include: (Baglan et al., 2018) (mosquitos); (Tahir et al., 2019) (spiders); (Farina et al., 2019; Tomé et al., 2020) (bees).

Also, like your earlier paragraphs, the paragraph that starts on line 84 is unfocused, in addition to being poorly referenced. If you’re going to discuss the half life of of glyphosate, you should be using more recent review papers such as (Singh et al. 2020) and (Van Bruggen et al. 2018). If you’re going to discuss how long it can remain in the top layer of soil, you should be citing all of the Laitinen papers. There is a rich body of literature discussing the impact of glyphosate on soil microbes and fungi. Look at Pochron et al. 2020 for a review of that.

But again, pick the point you want to make and make it in one paragraph. This paragraph wanders from run-off, to environmental concentrations, to the impact on soil microbes and its subsequent impact on plants, back to environmental concentrations, to the impact on algae, to the impact on bee microbiomes and fly microbiomes. No reader can make sense of the story you’re trying to tell.

In your final Introduction paragraph (line 96), you introduce the reader to a mushroom tyrosinase model with no explanation. You toss in a bunch of scientific names, without the common names, and you sketch out your experiment. At this point in the Intro, I should be able to determine how and why your experiment is important. It should tell me why you, the authors, think that glyphosate impacts melanin production. You don’t. I have no idea why you’re running these experiments.

Lastly, your Introduction and Discussion should bear some resemblance to each other. Your Discussion opens with AMPA, but AMPA is not discussed in your Intro.

For each of the outcome variables you measure in your Results (and you have many! All interesting!), you need to touch on those in your Intro. What impact do you expect to see in gut microbiomes, body mass, survivorship with and without infection, oocysts per midgut (or other measures of immune system, and all those things with mushrooms when you expose your models to glyphosate and AMPA? Your Intro should be a review of THAT literature. Since you have the insect apocalypse in your abstract, that should be in your Intro too.

**The Introduction needs to be completely revised**.

**Results:**

Given that PLOS Biology aims its papers are a more general readership than other more specialized journals, I recommend that you use fewer acronyms. PO, DQ, GLYPH, MBTH. Just use the real words. The readers don’t have to translate them continually and that improves readership.

I think that experiments 1-4 used some sort of mushroom model and that the remaining experiments use insect models. The results section might benefit from giving some sort of overview as to what experiments were run and what kind of model was used. (The Intro should cover that too, as well as tell us why.)

Much of the paragraph starting on line 105 doesn’t belong in Results. It belongs both in the Introduction and the Materials and Methods. Results sections should include only results.

Line 108, you have a sentence that begins with “First” but there is no second.

Actual results start on line 118. I am not an expert on methods of evaluating melanization. I can clearly see the dose dependent inhibition in Figure 1A, but I cannot understand this experiment given the Results and Figure 1A. A quick word-based sketch of what this experiment was supposed to do and show would benefit your readers. Something was tyrosinase mediated and something else was auto-mediated, but I can’t figure out your model. You should be able to communicate this model to other glyphosate specialists who are unfamiliar with mushrooms and melanization. Also, on line 120, you state that it appears that the inhibition in the reaction is due to inhibited background auto-oxidation. Please explain how you come to that conclusion. Why did you run the experiment with and without tyrosinase?

Section starting line 124: Clarifying as described in the paragraph immediately preceding this paragraph will benefit readers of your second experiment. Why do we care about the rate and level of DC produced? What does it tell us?

The experiment describe starting line 150 is really cool! The authors looked at non-phosphate analogs of glyphosate on DQ (melanin) production and compared them to phosphate-based analogs and glyphosate itself. Why did you home in on P? That should be in your introduction. Make non-experts care about this experiment. Make it clear that measuring DQ is a measure of melanin. Did you still use the mushroom model? Even adding a mushroom icon to your figures might help communicate your model more effectively. In 2F, consider putting the names of the compounds under their structures. Figuring out the name of each compound and finding it on the curves was nearly impossible.

Line 163, you state, “GLYPH and similar compounds inhibit melanin in a non-enzymatic fashion.” If I understand the experiment correctly, you selected phosphate-based compounds and non-phosphate-based compounds to determine which part of the glyphosate molecule drove the effect. (If I didn’t understand the experiment, please clarify it.) In your results, you need to describe the bimodal distribution—state that it’s based on P, which is pretty cool.

Line 214. You need one more sentence that interprets what the result means.

Line 339. Consider leading your results section with the insect models and then moving to the mushroom models.

Line 357: If you’re going to use AMPA in your experiments (and I agree you should!), you need to discuss it in your Introduction.

Line 358. You call out Fig 5 for the first time, after Fig 6. Noooo.

Lines 353-354 and 357-358 are highlights of this paper. They’re the reason I agreed to review this manuscript, and they’re the reason people will want to read the paper. You need to use all of your skills as writers to make your readers understand why those are interesting questions and why it is amazingly cool that you found your answers. Your Intro should be written so as to make me want to know what the impact of glyphosate and AMPA are on insect melanin production.

In Figure 6, you misspelled Fisher.

Lines 419-424. You need to get into the hormesis literature. My apologies. It isn’t fun.

Your experiments regarding insects are easier to understand and interpret than your results with the fungi. You need to set up the fungi experiments better. Something like, once we found these cool results with bugs, we wanted to find the mechanisms so we did this cool thing with a mushroom model. Because of this, please consider leading your results with the insect experiments and leading people to care about the mushroom results. It’ll make for a better read. Leading with the mechanism is slightly painful to read.

How close are the glyphosate doses you used in your insect experiments to those that they may encounter in nature? I mean when you injected the glyphosate.

**DISCUSSION:**

Line 485: what is *it*?

You have a paragraph on page 21 that covers almost an entire page. No one can read that. In fact, I recommend that you do through your Discussion and make sure all of your paragraph are no more than 1/3^rd^ of a page. In their current form, your paragraphs are driving away your readers, which will not improve the number of people who reference this awesome paper.

Your opening sentence of that paragraph (line 520) doesn’t make as much sense as one might hope. Your phrase, “which are necessary…” modifies *solution* but I think you want it to modify *free radicals* but maybe you want it to modify *inhibitors*. Line 524, the comma should be a period.

Lines 558-560. I object to using the same suite of references to make the point that glyphosate changes ecosystems by disturbing microbial populations AND inducing oxidative stress. Those should be two sets of references. ALSO, there are many, many papers that show that glyphosate has no impact or only a fleeting impact on ecosystems. (Most of those paper measure microbial biomass rather than community structure, but still, you should acknowledge that.) See for instance: Gornish, E.S., Franklin, K., Rowe, J. and Barberán, A., 2020. Buffelgrass invasion and glyphosate effects on desert soil microbiome communities. *Biological Invasions*, pp.1-11. Also, read the Discussion of Pochron et al. 2020 for the nuances involved in predicting the soil microbial response to glyphosate and Roundup exposure. The jury is still out on how bad glyphosate is for microbial systems.

Lines 580-581: Body weight does not always respond to exposure to contamination by decreasing (e.g. Pochron et al. 2018; 2019); in fact, sometimes organisms increase their body size in response to exposure to various contaminants, leading to an entire body of literature dedicated to hormesis, defined as a favorable biological response to exposure to toxins or other stressors (Agathokleous et al. 2019; Docea et al. 2019; Wang et al. 2018). Hormesis is most likely to occur at low dose exposures, and it doesn’t involve just glyphosate. The literature includes examples involving heavy metals and parasites. You are not alone in your vexing result regarding body weight.

Line 564: This paragraph is too long.

Line 608: You spend a lot of time talking about concentrations in run off. You’ve missed the literature showing that glyphosate and AMPA occur in our rain, soil, sediments. It would be nice if you could tie that universal contamination in to the ecosystem services delivered by insects. I know that no one will mourn the death of mosquitos, but they are good models for more beneficial insects. You can draw that line. I know nothing about your moth, but if its larva generally lives in the soil, then there is a whole body of literature about the ecosystem services delivered by soil-dwelling insects. People are going to read this paper because of the insect apocalypse angle. You’ve buried that lead in your text. I recommend that you bring it out front and center and make the mechanism the part of your manuscript that gives teeth to your experiments showing that glyphosate is bad for insects. Right now, this is paper is no fun to read. You can make it a lot better by focusing your Introduction and Discussion.
