# Supplementary material for: Glyphosate inhibits melanization and increases susceptibility to infection in insects
Source: PLoS Biol. 2021 May 12;19(5):e3001182. doi: 10.1371/journal.pbio.3001182 (PMC8115815; doi:10.1371/journal.pbio.3001182)
Supplement: Attachment — Submitted filename: PLOS review.docx [file pbio.3001182.s013.docx]

Reviewer: Sharon Pochron

**Abstract**

Line 1: Much improved title.

Line 14: Change to: Melanin, a black-brown pigment found throughout all kingdoms of life, has diverse biological functions including: UV protection, thermoregulation, oxidant scavenging, arthropod immunity, and microbial virulence.

Line 28: Change to: We showed that glyphosate acts as a synergistic antioxidant and disrupts the oxidation-reduction balance of melanization, elucidating the mechanism by which glyphosate inhibits melanization.

Overall, much improved.

**Introduction**

Line 39: Change to: Melanin, a black-brown pigment found throughout all kingdoms of life, has diverse biological functions including: UV protection, thermoregulation, oxidant scavenging, arthropod immunity, and microbial virulence.

Line 65: Change to: One ubiquitous chemical found in the environment is glyphosate, the most commonly used herbicide world-wide, which was previously shown to interfere with melanization in the fungus Cryptococcus neoformans (26).

Line 89: Put a return before the period to make a new para.

Line 127: This paragraph is not part of an Intro. It’s part of a Results or Conclusion. Remove it or move it.

I’d add chelation to the key words. I’d also add hormesis. You can remove Latin names since they’re in the Abstract.

Overall, much improved!

**Results**

Line 145: First comma should be a semicolon.

Line 160: Define PBS

Line 227: Missing a cap.

Line 320 and others regarding copper and glyphosate. I recommend citing:

Zhou, C.F., Wang, Y.J., Li, C.C., Sun, R.J., Yu, Y.C. and Zhou, D.M., 2013. Subacute toxicity of copper and glyphosate and their interaction to earthworm (Eisenia fetida). *Environmental Pollution*, *180*, pp.71-77.

Overall, much improved. Your paper is much easier to read and much easier to interpret.

**Discussion:**

Line 387: Your verb tenses do not agree. *Investigated* and *found*—not *find.*

Line 387:400. Great summary of your work. So much better.

Line 414: This paragraph is really interesting and important. I think I’d play up the public-health angle and see if you can work that point into the abstract or the key words. This is a packed paper, and you might not easily have room, but the fact that glyphosate exposure might make mosquitos more like to transmit plasmodium is really interesting and important.

Line 446: Again, really interesting results. Can you provide a list of the gut microbial species that increased and a list of species that decreased in the supplementary material?

Line 490: Again, you can reference that worm paper regarding copper and glyphosate here.

**Summary:**

In my opinion, this manuscript could be published with the minor revisions suggested above. The authors made all of the recommended changes in the previous review, and the manuscript is now readable and clear.
