# Supplementary material for: Glyphosate inhibits melanization and increases susceptibility to infection in insects
Source: PLoS Biol. 2021 May 12;19(5):e3001182. doi: 10.1371/journal.pbio.3001182 (PMC8115815; doi:10.1371/journal.pbio.3001182)
Supplement: Attachment — Submitted filename: Glyphosate Rebuttal Letter 02072021.docx [file pbio.3001182.s014.docx]

Dr. Ines Alvarez-Garcia

Senior Editor

PLOS Biology

February 7th, 2021

Dear Dr. Ines Alvarez-Garcia,

        	We appreciate the opportunity to revise and edit our previously submitted manuscript, “Glyphosate Inhibits Melanization and Increases Susceptibility to Infection in Insects.” Based on the reviewer comments we received, we have made changes to the manuscript, as itemized and detailed below. Overall, we have agreed with most of the reviewer’s suggestions for changes in the manuscript concerning sentence structure, grammar, and punctuation, and we have made additions where necessary.

To address Reviewer 1’s concerns about the statistical tests performed on the melanized nodule data (Figure 1D), we have analyzed the data using a nested non-parametric Mann-Whitney-Wilcoxon test to account for the variation between replicates. Regarding the concerns about pseudoreplication in our mosquito survival experiments (Figure 2C), we believe there was a misunderstanding of the data being presented. Wehave replaced our visualization with a classic Kaplan Meier survival curve, and have included a graphical representation of Hazard Ratios for each treatment group using a Cox Mixed Effects model, which takes into account both fixed and random effects (Figure 2D). We have also explained and justified our use of *ex vivo* studies to look at phenoloxidase activity, and we clarify that we also use *in vivo* studies to look at melanization within the *Galleria* during infection with *C. neoformans,* as seen in Figure 1 D-G.

To address Reviewer 2’s concerns about the relevancy of our data to “all insects,” we have included unpublished preliminary data in the following response in a *Drosophila melanogaster* fruit fly model indicating reduced melanization of glyphosate-fed flies following injury, and reference a recent manuscript showing that glyphosate causes changes in expression of melanization-related genes in *Apis cerana cerana* honeybees.  Together, this adds to four diverse species of insects, which suggests that generalization is possible.

As suggested by Reviewer 3, we have included two tables containing the relative abundance of the microbiome bacterial genera and classes following glyphosate exposure as Figures 3B,C. We have also corrected Figure 3C,D and Supplementary Figure 3D,E to remove an erroneous data point in the control group.

As suggested by the editor, we have changed the title of the manuscript to, “Glyphosate Inhibits Melanization and Increases Susceptibility to Infection in Insects.” We have changed the authorship order, and Dr. Nichole A. Broderick is now co-last author, as noted in the author byline and footnotes.

We would like to thank the reviewers and the editor for their helpful comments, suggestions, and feedback. We strongly believe that their help has improved the quality of our manuscript and the suggested experiments have strengthened our previous findings.

Sincerely,

Arturo Casadevall

Daniel Smith

**Reviewer 1:**

1. **Comment**: In this new submission, Smith and colleagues took into consideration previous comments made by the reviewers and presented a completely reorganized version of the manuscript (which is better in my opinion, and thanks to reviewer #3 comments, which provided insightful comments to restructure the manuscript). They also provided additional data, strengthening some of their findings.

**Response**: Thank you, and we appreciate your encouragement and support concerning our new findings. We feel that the manuscript was significantly improved by the modifications suggested by the reviewers and are grateful for those suggestions.

2. **Comment**: I am satisfied with their responses, and agree with their modifications and most of the statistical analyses provided. I have some additional questions and comments, but they are all minor considering the overall quality of the study.

**Response**: Thank you for the following comments, and we hope to address those comments and respond to the following questions satisfactorily.

3. **Comment:** One thing that called my attention while reading the manuscript for the second time was the performance of an ex vivo, instead of in vivo, experiment with G. mellonella hemolymph, or the incubation of mosquito homogenates with glyphosate to investigate melanization inhibition by glyphosate. I wonder why you chose this methodology, instead of performing a "more realistic" experiment by exposing the larvae/mosquito to glyphosate?

**Response:** For the phenoloxidase activity assays, we used the removed hemolymph of *Galleria (ex vivo)* and the homogenized mosquitoes based on previous studies. Direct measurement of the phenoloxidase kinetics is difficult to ascertain *in vivo.*

Melanization inhibition was assessed on reactions that occurred *in vivo* within *Galleria* (Figure 1D,E), since larvae treated with/without glyphosate were infected with *Cryptococcus neoformans* and then hemolymph was removed 24 hours later and nodules imaged under the microscope. As such, we believe these would be considered *in vivo* because the nodule formation and melanization (or lack thereof) being measured occurs within the living organism. These experiments mirror melanization immune response studies performed in *Anopheles* mosquitoes inoculated with Sephadex beads (Paskewitz and Riehle, 1994 <https://doi.org/10.1016/0145-305X(94)90002-7>; Warr et al. , 2006 [10.1016/j.ibmb.2006.07.006](https://doi.org/10.1016/j.ibmb.2006.07.006) ; Barreaux et al., 2017 [10.3791/55013](https://doi.org/10.3791/55013) . We have clarified that these experiments represent *in vivo* experiments in lines 164-168.

4. **Comment:** Why performing different statistical tests for similar experiments, such as the survival curve experiments presented in Fig 1C and Fig 2C?

**Response:** For Figure 1C we performed a Gehan-Breslow-Wilcoxon test, which weighs the earlier time points of a survival curve more than the later points. We chose this statistical test because we only treat the *Galleria* larvae with glyphosate once, five hours prior to infection, and thus expect the glyphosate’s effects to wear off and be stronger at the early time points of infection. For Figure 2C, we performed a log-rank (Mantel-Cox) test, which weighs each time point the same. We chose this test because the glyphosate treatment in mosquitoes is constant throughout the period measured and an evenly weighted test was more appropriate. We have added a sentence in the figure legend to clarify why the Gehan-Breslow-Wilcoxon test was performed in 1C.

5. **Comment:** Fig 1D,E: what's the concentration of glyphosate used here? Is it the same as for Fig 1C?

**Response:** For Figure 1C-E, the larvae are injected with 10 µl of 1 mM glyphosate in PBS. We have added a sentence in the figure legend to clarify this point

6. **Comment**: Fig1D FigS1G-I: here you pool data from three distinct experiments that do not show the same trend and do not consider that source of variation in your statistical analyses. I think you should give special attention to this.

**Response:** The data from the three replicates shows the same trends in terms of relative nodule sizes between the groups, however there is variation in their actual size between the glyphosate versus control infected groups in-between replicates. Additionally, the control (non-infected PBS treated) has roughly the same median particle size and roughly the same distribution, so we felt it would be appropriate to pool the three replicates. In order to account for the variations between the replicates have performed a nested non-parametric Mann-Whitney test to analyze statistical significance using the following R package: <https://github.com/douglasgscofield/nestedRanksTest> (Thompson et al 2014, <https://doi.org/10.1186/2051-3933-2-12>).

7. **Comment:** The end of lines 153-154 seems to be out of context, since you have only discussed the effects of glyphosate on G. mellonella by that point.

**Response:** Thank you for pointing out the confusion. We were referring to previous studies in fungi that were discussed in the introduction. To make this sentence clear, we changed the sentence to, “These data show that glyphosate inhibits melanization in insects similar to what has been previously shown in fungi (26), thus indicating that glyphosate interferes with major melanin-based processes in at least two kingdoms of life.” (now Lines 148-150).

8. **Comment**: Lines 46 and 244: since you opted to remove most abbreviations, I think it's unnecessary to abbreviate dopaquinone at these two instances, as well as DHI at lines 47 and 328

**Response**: We have removed the abbreviations as suggested. We have kept the dopaquinone abbreviation on Line 244 (now Line 251) because the DQ abbreviation Is used in the figure 4A for reasons of space and format.

9. **Comment:** Line 69: correct the sentence "responsible for amino acid synthesis IS many plants..."

**Response:** The sentence is corrected (Now Line 70).. Thank you!

10. **Comment:** Figure 2C - I could not find information regarding the way the experiment was conducted: were the mosquitoes kept in cages? If so, how many mosquitoes per cage? If there was more than one mosquito per cage and they were all counted for the survival curves, mosquitos in each cage should be considered as pseudoreplicates and this needs to be considered while performing the statistical analysis.

**Response:** During treatments, mosquitoes were kept in cardboard soup cups with a mesh lid in groups of 40 for each condition for the experiments shown in Figure 2C. This experiment was replicated three independent times. This configuration is considered standard in the field when measuring mosquito survival following treatments with compounds or a pathogen. We have included clarification of this in the figure legend and methods sections.

Thank you for pointing out the issue of pseudoreplication, in addressing this concern we learned that this is often not adequately addressed in the field and that it requires specific analysis to address. To address this issue we have changed the data visualization (Figure 2C) to that of a Kaplan-Meier survival curve, on which we performed the standard Mantel-Cox log rank test and corrected for multiple comparisons. We are also including a plot of the Hazard Ratios calculated from a Cox Mixed Effects Regression to account for the random effects of technical replicate as Figure 2D.

11. **Comment**: Figure 3D legend says that each treatment group represents 5 individual mosquito midguts, but the pcoa plot shows more than 5 dots for the control and treatment groups.

**Response**: Thank you for pointing the additional data points out! They were not meant to be included and represented a control condition. We have updated the figure with these removed.

12. **Comment:** Line 185: clarify what you mean when you mention "incubated"

**Response:** By, “incubated,” we mean that the glyphosate is added to the mosquito homogenate mixture and kept at 30°C while absorbance measurements are read. We have changed the sentence to read, “whole-body mosquito homogenate following the addition of glyphosate.” (Now lines 184-185).

13. **Comment**: Line 210: wrong figure citation: it should be Supplementary Fig. 2C, as in Line 214: wrong figure citation: it should be Supplementary Fig. 2D

**Response**: Thank you for pointing out this mistake. We have corrected the manuscript figure citations accordingly (Lines 216 and 220, respectively).

14. **Comment**: Figure 4 - Indicate the concentration of L-DOPA used in the experiments.

**Response**: We have added the L-DOPA concentration to the beginning of the results section that provides the Figure 4 results (Line 240). This information is also available in the Methods section (Lines 733, 765, 774, and 791)

**Reviewer 2:**

1. **Comment:** The authors have reorganized the manuscript so that the insect studies with one new data set are now first, and second is the biochemical characterization of how glyphosate affects the melanization reaction of a fungal tyrosinase. More background is presented for the insect work, and the writing and organization is more comprehensible. Still, as I wrote before, here are two very different types of experimental studies and no substantive basis for their linkage.

**Response:** We agree that the restructured manuscript, with the additional background information, as well as the inclusion of the additional data showing decreased melanization within nodules have strengthened our findings and made the manuscript stronger overall.

2. **Comment**: Fig. 1-3 present the data for the insect studies, and the effects of glyphosate are largely overly hyped in the Discussion for significance to global insect life and immunity to pathogens. Much ado about little, given that Galleria larvae are highly specialized honey bee pests and not representative of Lepidoptera in general and the overall non-significance of glyphosate treatments on Plasmodium infection and gut microbiota in Anopheles mosquitoes. There are lots more experimental results that appear to be solid for the glyphosate/fungal tyrosinase studies presented in Fig. 4-7. A link would be to show that an isolated insect tyrosinase/phenoloxidase involved in the melanization immune response acts similarly. Sure glyphosate at pharmacological/toxic levels affects "phenoloxidase" activity in unpurified larval hemolymph and ground up mosquitoes as likely would many other widely used chemicals for plant management.

**Response**: We appreciate the constructive feedback. We believe our data in two insect species separated by millions of years of evolution, the previously documented melanin inhibition in *C. neoformans* yeast, and our mechanistic data in the second part of the manuscript (Figures 4-7) suggests a broad glyphosate-based inhibition of melanization and DOPA polymerization. We do not have purified insect phenoloxidases, but the data in Figures 1A, S1A, and 2A serve a similar function, which use standard phenoloxidase activity assays to indicate that the glyphosate inhibits PO activity *in vitro.* Additionally, *Galleria mellonella,* while being specialized pests, are used as a model organism along-side other Lepidoptera model organisms *Manduca sexta* and *Bombyx mori,* the latter of which *Galleria* share 86% of its immune genes in common (Vogel et al. 2011. doi: 10.1186/1471-2164-12-308.).

3. **Comment:** It is toxic speculation that "glyphosate inhibits melanization and increases INSECT susceptibility to infection" as stated in the title and all too much so in the Discussion, maybe this can be weakly stated for Galleria larvae but certainly not all INSECTS!

**Response:** Our manuscript provides data for *Galleria* and *Anopheles.* Moreover, there is additional support that glyphosate impacts the melanin-based immune response in a recent study in *Apis cerana cerana* and we have preliminary data for *Drosophila melanogaster.*

A recently published manuscript dealing with *A. cerana cerana* honeybees (Zhao, et al 2020) shows that treatment with glyphosate-based herbicides increased expression of wounding and immune defense genes, including those related to melanization. The increased expression of melanization genes may be an indication of negative feedback as a result of glyphosate’s inhibition of melanization, or that the other surfactants and chemicals found In the commercial glyphosate formulations may trigger damage and/or immune defense systems. In addition, this paper identified a number of odorant binding proteins as being down-regulated by glyphosate treatment. We (Dr. Broderick) previously showed that in *D. melanogaster* and Tsetse fly that odorant binding proteins contribute to melanization and prophenoloxidase expression through impacts on crystal cells (Benoit *et al.* 2017).

While our manuscript includes data in *Galleria* and *Anopheles,* we have additional preliminary data in *Drosophila melanogaster* which we think are more appropriate for a follow up paper indicating that glyphosate weakens the melaninzation response in fruit flies. According to this data performed in the Broderick Lab (attached below), glyphosate-drugged food decreases the melanin-based wound repair response in *Drosophila* following sterile wounding. This indicates a third insect species in which glyphosate weakens the melanization reaction. We have edited the manuscript to tone down the language to state that our data in two evolutionarily distinct insect species, as well as a fungal tyrosinase, only suggests this effect is would occur broadly in insects.

Given evidence that the herbicide affects four insect species and that our work establishes mechanistic causality by showing that glyphosate inhibits melanization, which is a critical host defense mechanism, we think there is a strong case that our findings represent a general phenomenon. However, we have heeded the editor recommendation to soften the conclusions by modifying some discussion sentences. Additionally, in accordance with your feedback and as suggested by the editor, we have changed the manuscript title to, “Glyphosate inhibits melanization and increases susceptibility to infection in insects."

**A.**

**Melanized Clot	No Melanized Clot**

**B.**

**Glyphosate feeding reduces the melanotic wounding response of adult *Drosophila melanogaster* following sterile injury*.*** Upon adult eclosion, flies were placed on Broderick Lab standard food (Lesperance and Broderick 2020) to which a low, medium, and high level of glyphosate has been added. Flies were maintained on the glyphosate impregnated food prior to experimental assays. To examine the impact to the cuticular melanization wounding response, adult flies were pricked with a sterile minuten insect pin in the thorax (**A**). The degree to which a melanotic clot developed was assessed visually on individual flies (after six hours and ranked as none (A, right panels), medium, or high (A, left panels). The mean percent of flies (two replicates of n=20 flies per treatment) in each category is shown (**B). Data and figure courtesy the Broderick laboratory.**

Reviewer 3:

Abstract

1. **Comment**: Line 1: Much improved title.

**Response**: Thank you!

2. **Comment:** Line 14: Change to: Melanin, a black-brown pigment found throughout all kingdoms of life, has diverse biological functions including: UV protection, thermoregulation, oxidant scavenging, arthropod immunity, and microbial virulence.

**Response:** Thank you for the suggestion, we have made the edits as suggested, now Lines 15-17.

3. **Comment**: Line 28: Change to: We showed that glyphosate acts as a synergistic antioxidant and disrupts the oxidation-reduction balance of melanization, elucidating the mechanism by which glyphosate inhibits melanization.

**Response**: Thank you. We have changed this line to, “Our results show that glyphosate’s mechanism of melanin inhibition involves antioxidant synergy and disruption of the reaction oxidation-reduction balance.” Now beginning on Line 30.

4. **Comment:** Overall, much improved.

**Response:** Thank you, we believe the suggestions and feedback during the previous round of peer review have helped improve the quality of the abstract.

Introduction

5. **Comment**: Line 39: Change to: Melanin, a black-brown pigment found throughout all kingdoms of life, has diverse biological functions including: UV protection, thermoregulation, oxidant scavenging, arthropod immunity, and microbial virulence.

**Response**: We have edited the sentence to read, “Melanin, a black-brown pigment found in all biological kingdoms, is produced through a series of oxidation and reduction reactions.” (now Lines 40-41)

6. **Comment**: Line 65: Change to: One ubiquitous chemical found in the environment is glyphosate, the most commonly used herbicide world-wide, which was previously shown to interfere with melanization in the fungus Cryptococcus neoformans (26).

**Response**: Thank you, we have made the suggested edit. Now lines 66-68.

7. **Comment**: Line 89: Put a return before the period to make a new para.

**Response:** Thank you for the suggested edit. We have made the changes as requested. Now line 90.

8. **Comment**: Line 127: This paragraph is not part of an Intro. It’s part of a Results or Conclusion. Remove it or move it.

**Response**: We have removed the paragraph, as we believe this paragraph is redundant with similar paragraphs of the results and conclusion.

9. **Comment:** I’d add chelation to the key words. I’d also add hormesis. You can remove Latin names since they’re in the Abstract.

**Response:** We have added hormesis, antioxidant, synergism as key words but we have not removed the Latin names., which we leave to the discretion of the journal and editor.

10. **Comment**: Overall, much improved!

**Response**: Thank you!

Results

11. **Comment**: Line 145: First comma should be a semicolon.

**Response**: Comma replaced with a semicolon as suggested.

12. **Comment**: Line 160: Define PBS

**Response:** The sentence has been edited to include the definition of PBS before introducing the acronym. Now Line 156.

13. **Comment** Line 227: Missing a cap.

**Response:** Sentence edited to capitalize “glyphosate”

14. **Comment** Line 320 and others regarding copper and glyphosate. I recommend citing:
    Zhou, C.F., Wang, Y.J., Li, C.C., Sun, R.J., Yu, Y.C. and Zhou, D.M., 2013. Subacute toxicity of copper and glyphosate and their interaction to earthworm (Eisenia fetida). Environmental Pollution, 180, pp.71-77.

**Response:** Thank you for the paper suggestion! We have included a sentence following this line saying, “Similar results have been previously seen in *Eisenia fetida* earthworms exposed to high copper conditions in soil. Glyphosate contamination of copper-rich soil reduced the detrimental effects of the metal’s toxicity, presumably due to the glyphosate’s copper chelation properties.” Now Lines 329-332.

15. **Comment:** Overall, much improved. Your paper is much easier to read and much easier to interpret.

**Response:** Thank you! We greatly appreciate the constructive feedback and the help!

Discussion:

16. **Comment:** Line 387: Your verb tenses do not agree. Investigated and found—not find.

**Response:** Thank you! Edit has been made to correct the tense. . (Now lines 398-399)

17. **Comment:** Line 387:400. Great summary of your work. So much better.

**Response:** Thank you! We believe the constructive feedback has been very helpful in improving the clarity and quality of our manuscript.

18. **Comment:** Line 414: This paragraph is really interesting and important. I think I’d play up the public-health angle and see if you can work that point into the abstract or the key words. This is a packed paper, and you might not easily have room, but the fact that glyphosate exposure might make mosquitos more like to transmit plasmodium is really interesting and important.

**Response:** Thank you! We have added “vector biology” to the keywords to make it easier for people to find when looking for information about disease transmission in insects. We have added a sentence at the end of this paragraph stating, “Overall our results raise concerns for public health and malaria control initiatives in regions in which malaria is endemic and where there is increasing use of glyphosate, including areas of Latin America, Sub-Saharan Africa, and Asia. “ Additionally, the sentence beginning on Line 559 has been edited to include reference to the public health implications. It now reads, “This suggests protean consequences for human health ranging from ecosystem disruption to altered vector competency of lethal human pathogens and increased malaria transmission in endemic regions that use glyphosate-based herbicides in agriculture.”

19. **Comment**: Line 446: Again, really interesting results. Can you provide a list of the gut microbial species that increased and a list of species that decreased in the supplementary material?

**Response**: Unfortunately, the resolution of these analyses can only reliably determine the genus of bacteria, and for some, only the class. We have included two tables in Figure S3 (Figures S3B,C) with the genus and class-specific specific breakdown of relative bacterial abundance.

20. **Comment:** Line 490: Again, you can reference that worm paper regarding copper and glyphosate here.

**Response:** Thank you for the suggestion. We have included the following sentence referring back to this paper, “It appears that glyphosate, possibly through chelation, acts as a “buffer” of copper ions and can reduce the metal’s harmful effects, similar to previous findings concerning the toxicity of high-copper soil to earthworms following glyphosate treatment.” Now Lines 516-519.

Summary:

21. **Comment:** In my opinion, this manuscript could be published with the minor revisions suggested above. The authors made all of the recommended changes in the previous review, and the manuscript is now readable and clear.

**Response:** Thank you! We appreciate the support of our manuscript as well as the suggestions and the feedback provided during these two rounds of revisions.
