# Supplementary material for: Glyphosate inhibits melanization and increases susceptibility to infection in insects
Source: PLoS Biol. 2021 May 12;19(5):e3001182. doi: 10.1371/journal.pbio.3001182 (PMC8115815; doi:10.1371/journal.pbio.3001182)
Supplement: Attachment — Submitted filename: Glyphosate Response Letter 03012021.docx [file pbio.3001182.s015.docx]

Dr. Ines Alvarez-Garcia

Senior Editor

PLOS Biology

March 2^nd^, 2021

Dear Dr. Ines Alvarez-Garcia,

        	We appreciate the consideration of our submitted manuscript, “Glyphosate Inhibits Melanization and Increases Susceptibility to Infection in Insects.”

Following PLOS Biology’s guidelines and requirements as you have stated, we organized and uploaded the data underlying our figures in the free and accessible online repository Figshare. We have included the link to the deposited data in the “Data Availability” section of the manuscript, as well as a supplementary table (Supplementary Table 1), which includes DOI links to each figure’s underlying data on Figshare. We have included where to find the data links in all of the figure legends.

We have verified that the references in this manuscript are correct, and that none to the best of our knowledge have been retracted.

Best,

Arturo Casadevall
